# Long-term exposure to exogenous phthalate, masculinity and femininity trait, and gender identity in children: a Chinese 3-year longitudinal cohort study

**DOI:** 10.1186/s12940-023-01031-5

**Published:** 2023-11-28

**Authors:** Jieyu Liu, Di Gao, Huan Wang, Yanhui Li, Manman Chen, Qi Ma, Xinxin Wang, Mengjie Cui, Li Chen, Yi Zhang, Tongjun Guo, Wen Yuan, Tao Ma, Jianuo Jiang, Yanhui Dong, Zhiyong Zou, Jun Ma

**Affiliations:** 1 Institute of Child and Adolescent Health, School of Public Health, National Health Commission Key Laboratory of Reproductive Health, Peking University, Xueyuan Road 38, Beijing, China; 2 Department of Obstetrics and Gynecology, Peking University First Hospital, Beijing, China; 3 School of Nursing, Peking University, Beijing, China; 4 School of Public Health and Management, Ningxia Medical University, Yinchuan, China

**Keywords:** Phthalates, Gender identity, Masculinity, Femininity, Children

## Abstract

**Background:**

Phthalate esters (PAEs) are known to have hormone-like properties, and there is a growing trend of children expressing a gender identity different from assigned sex. However, there has been limited research in the potential links between PAEs exposure and gender identity.

**Methods:**

A total of 571 children (278 boys) completed the follow-up from Oct 2017 to Oct 2020 in Childhood Blood Pressure and Environmental Factors (CBPEF) cohort in Xiamen, China. Urinary PAE metabolites were measured at three time of visits using ultraperformance liquid chromatography-tandem mass spectrometry. The Children’s Sex Role Inventory scale was used to assess gender identity (masculinity, femininity, androgyny and undifferentiated), and Tanner definition was used to define puberty timing. Generalized linear models and log-binomial regression were used to assess the relationships between PAEs exposure, gender trait scores and gender identity.

**Results:**

Overall, the concentration of most PAEs in more than 90% of participants was above the limit of detection values. In visit 1, there were 10.1% boys with femininity and 11.3% girls with masculinity; while these figures increased to 10.8% and 12.3% during follow-up, respectively. Early puberty onset accounted for 24.8% and 25.6% among boys and girls. Long-term exposure to mono-2-ethylhexyl phthalate (MEHP) (β = 1.20, 95%CI = 0.13, 2.28), mono-2-ethyl-5-hydroxyhexyl phthalate (MEHHP) (β = 1.25, 95%CI = 0.22, 2.28) and mono-2-ethyl-5-oxohexyl phthalate (MEOHP) (β = 1.40, 95%CI = 0.24, 2.56) was associated with the increased differences of femininity trait scores in boys who enter puberty earlier, prolonged exposure to di(2-ethylhexyl) phthalate (DEHP) might also have such a positive impact (β = 1.38, 95%CI = 0.36, 2.41). For gender identity, persistent exposure to low molecular weight phthalates (LMWP) was negatively associated with undifferentiated type among boys entering puberty earlier (RR = 0.18, 95%CI = 0.05, 0.75, *P* < 0.05), and most of the PAE metabolites exposures showed risk ratios > 1 for their femininity.

**Conclusion:**

Long-term exposure to PAEs increase the femininity trait scores in boys with early onset of puberty. Although the mechanisms remain to be determined, environmental pollution might have subtle, yet measurable effects on childhood gender identity. Reducing these chemicals exposure has important public implications on gender development.

**Supplementary Information:**

The online version contains supplementary material available at 10.1186/s12940-023-01031-5.

## Introduction

Used in a variety of consumer products, phthalate esters (PAEs) are a group of chemicals that are ubiquitous in the environment. These chemicals have been identified as endocrine-disrupting chemicals (EDCs) and possess anti-androgenic properties, which can interfere with hormone metabolism [1]. As a result, they pose a significant risk to human health [2]. Exposure to PAE metabolites may affect specific pubertal development characteristics [3], [4], and was associated with earlier puberty in boys with normal weight [5].

Sexual and reproductive health and rights (SRHR) are essential for the overall health of individuals, economic development, and the well-being of humanity [6]. Gender, a broad term that encompasses social and cultural factors in addition to biological sex, plays a significant role in shaping cognition, behaviors, and social relationships. Gender is also a social determinant of cardiovascular health, and implementing gender equality strategies could help reduce the burden of cardiovascular diseases at the population level [7]. Current research distinguishes between three primary dimensions of gender: gender identity, gender roles and gender norms (i.e. societal constructs that lead to shared ideas about what constitutes masculinity and femininity). Gender identity refers to the sense of self as male or female, or neither [8], and is a central construct of psychosocial development and mental health. About 5% of young adults in the U.S. said their gender was different from their sex assigned at birth [9], with a considerable number identifying as non-binary notions of gender (male vs. female) [10]. Children and adolescents who had gender dysphoria (a discrepancy between natal gender and gender identity) may experience depression, anxiety and social stigmatization [11].

The process of sexual differentiation is complex and influenced by multiple factors, including genetic, biological, and environmental determinants. Hormonal changes in the fetal environment may have effects on sexually-dimorphic behaviors [12], and testosterone during early infancy may contribute to neurobehavioral sexual differentiation [13]. In addition, play behaviors in children are accepted methods for determining gender identity and are critical factors in diagnosing gender dysphoria [14]. Limited evidence suggested that maternal urinary concentrations of PAE metabolites during pregnancy may be associated with less masculine gender-related play behaviors and decreased composite scores among preschool-aged boys [15]–[17]. In contrast, higher maternal urinary concentrations of MEP may be associated with more typical gender-related play behaviors in both males and females, indicating a possible link between maternal PAEs exposure and gender-related behaviors [16]. However, these studies have mainly focused on gestational and prenatal exposure to PAEs, and the connection between childhood PAE metabolites exposure and gender identity has yet to be investigated.

Given the fact that several PAEs had potential anti-androgenic activity and no study explored the associations between PAEs, masculinity/femininity trait and gender identity, we aimed to investigate the associations between long-term PAEs exposure and gender identity in children, based on a longitudinal cohort study with repeated measurement of urine.

## Materials and methods

### Study participants

From May 2017 to October 2020, Childhood Blood Pressure and Environmental Factors (CBPEF) cohort was conducted in Xiamen, China. Initially, a pilot study was conducted in May 2017, and four 9-Year Schools covering both primary (grade 1 to 6) and middle (grade 7 to 9) school stage were randomly selected, including a total of 4923 students of grade 2 to 4. Children with severe organ diseases (such as heart, lung, liver, or kidney conditions), abnormal physical development, or signs of puberty (such as genital development in males or breast development in females indicating entry into Tanner Stage 2) were excluded from the study. Subsequently, in October 2017, we conducted a baseline survey, recruiting students from two schools covering both primary and middle school stage (n = 1430), and excluding those who refused to participate in the tests involving urine collections. A medical examination was conducted to ensure that the participants met the inclusion criteria.

**Fig. 1:**
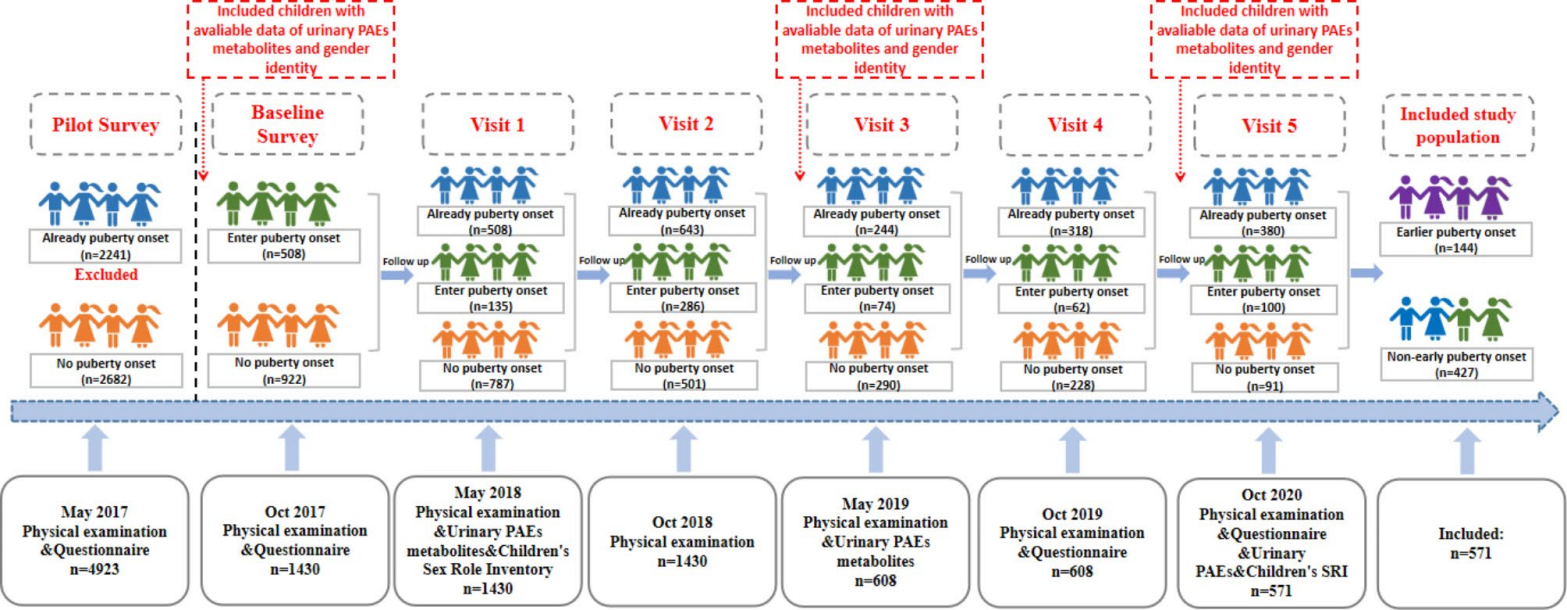
The study design and study population selection during consecutive years

In the first wave of visit in May of 2018 (visit 1), all included participants were asked to submit morning void urine samples and demographic questionnaires (n = 1430, of which 922 children did not enter puberty). In visit 3 and visit 5, we also asked children to submit the first-morning void urine samples. In addition, the anthropometric measurements were done and the level of sexual development was scored in each visit of surveys. In visit 1 and 5, we included children with available data of Children’s Sex Role Inventory. After the follow-up survey, we included 571 children of grade 2 to 4 with available urine samples, with boys and girls of mean age of 9.53 ± 0.59 years and 8.49 ± 0.55 years. The detailed process of study design and population selection during consecutive years is presented in Fig. 1. Our study was evaluated and approved by Medical Ethical Committee of Peking University Health Science Center (IRB00001052-21084), and we also got the written informed consent from all included students and their parents.

### Phthalates metabolite measurements

The urine samples were collected by healthcare providers using polypropylene cups to collect the first-morning void samples. These samples were then stored in polypropylene containers to prevent contamination with other PAEs from environmental sources. The laboratory followed the operational procedures from the National Health and Nutrition Examination Survey and developed a standardized methodology for analyzing PAEs metabolites using ultra-high performance liquid chromatography-triple quadrupole mass spectrometry (UPLC-MS/MS). All urine samples were stored at -80 °C until testing. To ensure the integrity of the results, two UPLC-MS/MS water blank samples were set up to check for any pollution caused by sample preparation. Additionally, other plastic laboratory consumables used in the process were made of polypropylene products that did not contain PAEs. The standards solutions used for analysis included seven PAEs metabolites: mono-ethyl phthalate (MEP), mono-methyl phthalate (MMP), mono-n-butyl phthalate (MnBP), mono-2-ethylhexyl phthalate (MEHP), mono-2-ethyl-5-hydroxyhexyl phthalate(MEHHP), mono-iso-butyl phthalate (MiBP), and mono-2-ethyl-5-oxohexyl phthalate (MEOHP).

In the present study, the limit of detection (LODs) of these PAE metabolites is shown in Table S1. The PAEs concentrations below LODs used a value of LODs divided by the square root of 2. PAEs are divided into two groups according to their molecular weight and parent PAEs: high molecular weight phthalates (HMWP, such as di-(2-ethylhexyl) phthalate (DEHP) including MEHP, MEOHP, and MEHHP) and low molecular weight phthalates (LMWP, including MMP, MEP, MnBP, and MiBP). We calculated all the molar sum of each metabolite as the summary measures of PAEs (∑PAEs), all the molar sum of each low molecular weight phthalate metabolite as ∑LMWP, and all the molar sum of each high molecular weight phthalate metabolite as ∑DEHP. To obtain creatinine-adjusted PAE metabolites, each PAE metabolite concentration was divided by the ratio of observed creatinine to expected creatinine [18]. Figure S1 showed the appropriate scaled normal density for log-transformed values of PAEs. In addition, exposure to PAEs metabolites was classified into two groups in each visit, that was, high exposure group (PAEs exposure ≥ P50) and low exposure group (PAEs exposure < P50) according to their sex-specific 50th percentiles, and the Intraclass Correlation Coefficients (ICC test) in three visits are shown Table S2. Further, we combined the group at each visit, if children were exposed to PAEs metabolites of < P50 for all three visits, we categorized these children into persistent low (PL) exposure group, if they were exposed to PAEs metabolites of ≥ P50 for all three visits, they were grouped into persistent high (PH) exposure group, otherwise they belonged to fluctuated exposure group (This referred to the situation where PAE exposure was below the 50th percentile during one visit and at or above the 50th percentile during the other two visits, or exposure was at or above the 50th percentile during one visit and below the 50th percentile during the other two visits).

### Definition of gender identity and early puberty onset

The questionnaires for gender identity were conducted in visit 1 and the visit 5. Children would fill in children’s questionnaire by themselves instructed by their parents or class teacher. In addition, trained project members interpreted all the questionnaires in detail. Appropriate guidance would be given by these project members as effectively as possible. The Chinese version of Children’s Sex Role Inventory (CSRI) scale verified by Chinese scholars was used to assess gender role for children [19], with great reliability determined acceptable by the Cronbach’s alpha coefficients of 0.83 and 0.77 for the masculinity and the femininity sub-scales, respectively. It is a 52-item standardized psychometric self-report instrument consisting 17 masculine options, 15 feminine options, and 20 neutral options (Table S3). Each item bases on very conforming, most conforming, partially conforming, non-compliant options and the corresponding scores are 4 − 1 points. Masculinity trait score (M) = total masculinity trait score/17; femininity trait score (F) = total femininity trait score/15. In visit 1, the median masculinity and femininity trait scores of boys were 2.82 and 2.87 respectively, and the median masculinity and femininity trait scores of girls are 2.88 and 3.13 respectively. Hence, the gender identity of boys was categorized into masculinity (M ≥ 2.82, F < 2.87), femininity (M < 2.82, F ≥ 2.87), androgyny (M ≥ 2.82, F ≥ 2.87), and undifferentiated (M < 2.82, F < 2.87). Similarly, the gender role of girls was classified into masculinity (M ≥ 2.88, F < 3.13), femininity (M < 2.88, F ≥ 3.13), androgyny (M ≥ 2.88, F ≥ 3.13), and undifferentiated (M < 2.88, F < 3.13). In visit 5, the median masculinity and femininity trait scores of boys were 2.76 and 2.87 respectively, and the median masculinity and femininity trait scores of girls are 2.82 and 3.07 respectively. Hence, the gender identity of boys was categorized into masculinity (M ≥ 2.76, F < 2.87), femininity (M < 2.76, F ≥ 2.87), androgyny (M ≥ 2.76, F ≥ 2.87), and undifferentiated (M < 2.76, F < 2.87). Similarly, the gender role of girls was classified into masculinity (M ≥ 2.82, F < 3.07), femininity (M < 2.82, F ≥ 3.07), androgyny (M ≥ 2.82, F ≥ 3.07), and undifferentiated (M < 2.82, F < 3.07).

The measurement of Tanner stage of pubertal development and pubic hair in children was conducted by four fixed physicians (two males and two females). Children of the same sex were asked to take off all clothes (except for underpants) in a warm and completely enclosed room, and the specific Tanner stage of secondary sexual characteristics and pubic hair were determined based on the Tanner stage pictures and testicular development models. When the actual stage was between 2 stages, the less developed stage was recorded; when the stage of breasts or testis on two sides was different, the more developed stage was recorded. Pubertal stage I was defined as pre-puberty, pubertal onset was determined as girls’ breast or pubic hair and boys’ testis volume or pubic hair attained Tanner stage II [20]. Among all the children with determined age of pubertal onset, we recorded the age of entering Tanner stage II as the puberty onset age. Then, the early onset of puberty was defined as children whose puberty onset age earlier than the first quartile among the children of the same sex [21], that was, the puberty onset age earlier than 10.33 years in boys and 8.97 years in girls in the current study.

### Physical measurement and questionnaire

Using a standardized protocol, children’s body weight (kg) was measured to the nearest 0.1 kg, whereas height (cm) was measured to the nearest 0.1 cm in bare feet. Weight status categories among participants were determined by body mass index (BMI), calculated as body weight (kg) divided by height (m) squared (kg/m^2^) using the criteria set by the Working Group on Obesity in China. Overweight and obesity was defined as BMI ≥ 85th percentile [22].

Self-administrated questionnaires were distributed to collect the potential con-founders which were filled out by one of their parents. The child’s basic demographic information were contained in the questionnaire, including sex, age, single-child status, parental occupation, and monthly household income. Single-child status was classified into “yes” or “no”. Parental occupation was grouped into “civil servant, businessman/company employee/worker, housework/unemployed/laid-off/retirees”. The monthly household income of one family is the total income of all family members, and then was determined as follow: < 5000 Chinese Yuan (CNY), 5000–12,000 CNY, ≥ 12,000 CNY, and unknown or refuse to answer.

### Statistical analysis

The traditional analysis adopted the classical statistical analysis method including the Student t-test and Chi-square (χ^2^) test. Due to the skewness distribution for sex hormones and PAEs original values, we calculated their log conversion values. The appropriately scaled normal density was drawn for log conversion values. The original values of urinary PAEs, and their log conversion values were used to analyze their geometric means distribution. Generalized additive models (GAMs) were used to fit smooth curves and establish linear or non-linear relationships between PAEs exposure and gender trait scores in each single visit, and a series of generalized linear models (GLMs) were used to assess the relationships between log-transformed PAEs exposure and gender trait scores separately in boys and girls. We also investigated the linkage between long-term PAEs exposure and the differences of gender trait scores between visit 1 and visit 5. The model was adjusted for age, single-child status, monthly household incomes, parents’ occupation and gender trait scores in visit 1. Then, to clarify the associations between PAEs and gender identity in children, we calculated the adjusted Risk Ratios (ARR) and 95% confidence intervals (CIs) using log-binomial regression models with PAEs separately as independent variables, as the proportion of the gender identity was greater than 10%, odds ratios give biased estimates of prevalence ratios. The results of Goodness-of-Fit test are shown in Table S4. Some potential confounders were adjusted in the model including age, single-child status, monthly household incomes, parents’ occupation and gender identity in visit 1. Previously, we found that the long-term PAEs exposure was positively related with early pubertal onset [23], to avoid the potential influence of early pubertal onset on such associations, sensitivity analyses were conducted when we only restricted to children with early pubertal onset during the follow-up. Significance tests were evaluated at the 0.05 level, using two sided tests. All data cleaning and statistical analyses were conducted using SAS 9.4 (SAS Institute Inc., NC, USA).

## Results

### Characteristics of study population

The characteristics of the children in the baseline study and subsequent survey visits were presented. A total of 753 boys and 677 girls completed the baseline survey, with an average age of 9.00 years (SD = 3.44) for boys and 8.03 years (SD = 0.77) for girls. Additionally, 40.9% of boys and 45.8% of girls came from families with a household monthly income exceeding 12,000 CNY (Table S5). A total of 571 children including 278 boys completed the follow-up surveys (Table 1). The average age of boys and girls was 9.53 ± 0.59 and 8.49 ± 0.55 years. Approximately 31.3% of boys and 29.0% of girls were only children in their families. Moreover, about half of the children’s mothers and fathers were civil servants. There were 24.8% and 25.6% boys and girls had early puberty onset in the final visit. The concentrations of MMP, MEP, MiBP, and MEHHP were above the LOD values in more than 90% of the participants in total (Table S6) and across three PAEs exposure groups Table S7).

**Table 1:** Characteristic of the follow-up pediatric population from the longitudinal cohort study in China

Follow-up population (n = 571)	Boys (n = 278)	Girls (n = 293)	*P-value*
Age, year, mean ± SD	9.53 ± 0.59	8.49 ± 0.55	< 0.001
Single-child status, n(%)	87 (31.3%)	85 (29.0%)	0.451
*Maternal occupation*^a^, *n(%)*			0.066
Civil servant	143 (51.4%)	144 (49.1%)	
Businessman/Company employee/Worker	83 (29.9%)	111 (37.9%)	
Housework/Unemployed/laid-off/Retirees	52 (18.7%)	37 (12.6%)	
Missing data	0 (0.0%)	1 (0.3%)	
*Paternal occupation*^a^, *n(%)*			0.321
Civil servant	132 (47.5%)	126 (43.0%)	
Businessman/Company employee/Worker	105 (37.8%)	132 (45.1%)	
Housework/Unemployed/laid-off/Retirees	39 (14.0%)	34 (11.6%)	
Missing data	2 (0.7%)	1 (0.3%)	
*Monthly household income, n(%)*			0.101
< 5000 CNY	4 (1.4%)	3 (1.0%)	
5000–12,000 CNY	32 (11.5%)	33 (11.3%)	
≥ 12,000 CNY	109 (39.2%)	144 (49.1%)	
Unknown or refuse to answer	133 (47.8%)	113 (38.6%)	
Earlier puberty onset during follow-up, n(%)	69 (24.8%)	75 (25.6%)	0.831
**Gender trait scores**			
**Children’s gender trait scores in visit 1, median (IQR)**			
Masculinity trait scores	2.82 (0.82)	2.88 (0.76)	0.156
Femininity trait scores	2.87 (0.80)	3.13 (0.67)	< 0.001
**Children’s gender trait scores in visit 5, median (IQR)**			
Masculinity trait scores	2.76 (0.71)	2.82 (0.76)	0.163
Femininity trait scores	2.87 (0.67)	3.07 (0.67)	0.737
**Changes of children’s gender trait scores, mean ± SD**			
Changes of Masculinity trait scores	0.13 ± 1.08	-0.06 ± 0.59	< 0.001
Changes of Femininity trait scores	0.18 ± 1.22	-0.07 ± 0.59	< 0.001

^a^For the employment engaged in more than one occupation, we indicated the occupation with the longest working period. CNY, Chinese yuan

### Sex-specific distribution of gender trait scores and percentage of each gender identity type

As shown in Figure S2, there was a notable rise in femininity trait scores among boys from visit 1 to visit 5 (*P* < 0.01). In visit 1, there were 10.1% boys with femininity and 11.3% girls with masculinity; while these figures increased to 10.8% and 12.3% in visit 5, respectively. Conversely, the percentage of femininity type decreased in girls (*P* < 0.05), while the undifferentiated type increased during follow-up (*P* < 0.01). Since the included children were younger, the percentages of undifferentiated type in both sexes were all approximately over 40.0%, respectively.

### Associations between phthalate exposure and gender trait scores

**Fig. 2:**
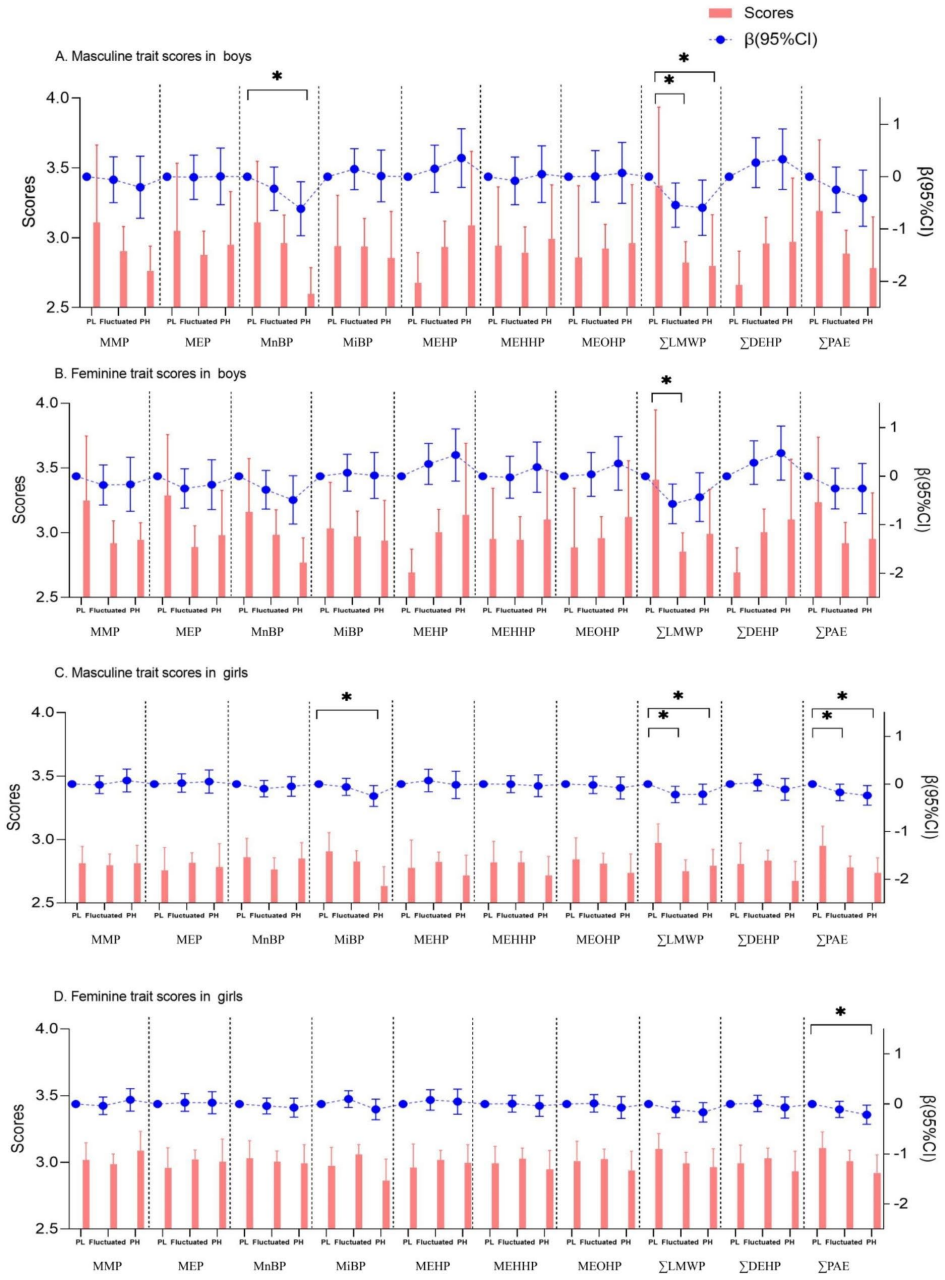
Linear associations between long-term PAEs exposure and the masculinity trait and femininity trait scores in children. (*95%CI did not contain 0 referred to *P* < 0.05; PL group was considered as reference group)

Overall, results showed linear associations between PAEs exposure and gender trait scores in visit 5 (*non-linear P* > 0.05, Table S8 and Figure S3). After controlling for the gender trait scores in visit 1, persistent high exposure to LMWP was significantly associated with decreased masculinity trait scores in visit 5 in boys (β=-0.59, 95%CI=-1.12, -0.07) and girls (β=-0.21, 95%CI=-0.42, 0.00), respectively, with the significant decrement of masculinity trait scores across PAEs groups in parallel (both *P* < 0.05) (Fig. 2).We further divided the population according to pubertal onset timing. Across different PAEs metabolites exposure group, the masculinity trait scores of boys with early pubertal onset decreased significantly in the group of persistent high exposure to LMWP (*P* = 0.014) (**Figure S4**). However, the increasing trends of femininity trait scores across exposure groups were not detected among boys without earlier onset of puberty (**Figure S5**).

**Fig. 3:**
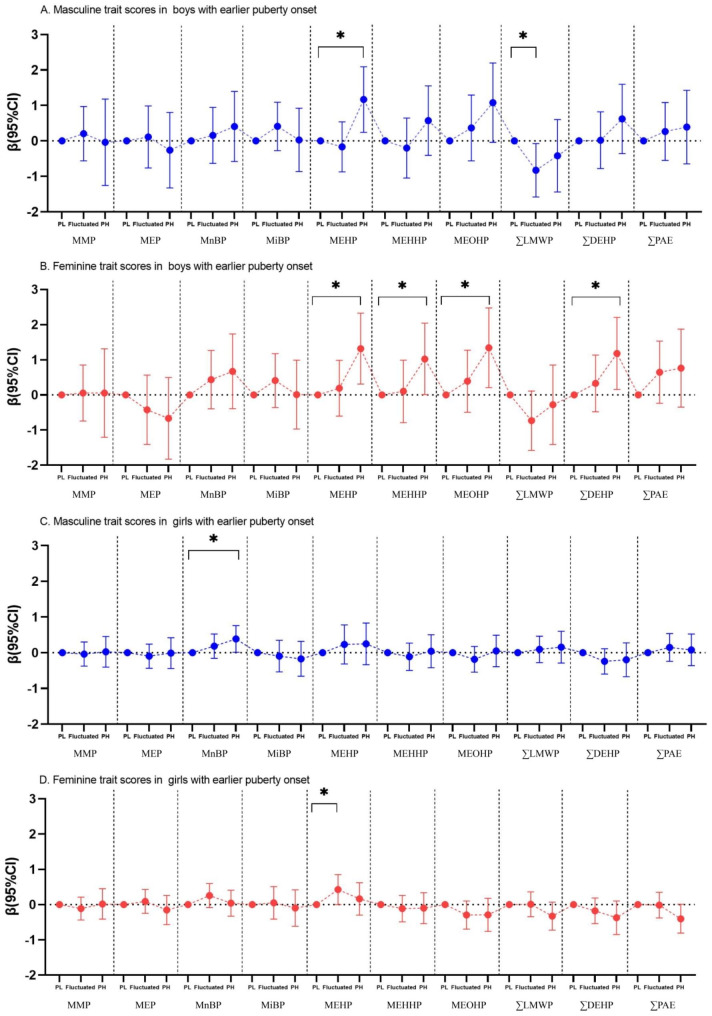
Linear associations between long-term PAEs exposure and the masculinity trait and femininity trait scores in children with early onset of puberty. (*95%CI did not contain 0 referred to *P* < 0.05; PL group was considered as reference group)

Figure 3 shows the associations between long-term PAEs exposure and the masculinity and femininity trait scores in visit 5 in children with early onset of puberty, additionally after adjusting for the gender trait scores in visit 1. Long-term exposure to MEHP was significantly related with increased scores of femininity trait (β = 1.32, 95%CI = 0.31, 2.32) and masculinity trait (β = 1.17, 95%CI = 0.24, 2.09) in boys with earlier puberty onset, and exposure to MEHHP (β = 1.02, 95%CI = 0.01, 2.04) and MEOHP (β = 1.34, 95%CI = 0.21, 2.48) were also related with the increased scores of femininity trait. Slightly increased scores of masculinity trait in girls with early onset of puberty were also detected when they were exposed to persistent high MnBP (β = 0.39, 95%CI = 0.01, 0.76). However, such increasing trends of femininity or masculinity trait scores were not detected among children without early pubertal onset (Figure S6), but long-term exposure to MnBP was associated with decreased masculinity trait scores in boys (β=-0.91, 95%CI=-1.51, -0.31).

### Associations between phthalate exposure and the difference of gender trait scores from the visit 1 to visit 5

**Fig. 4:**
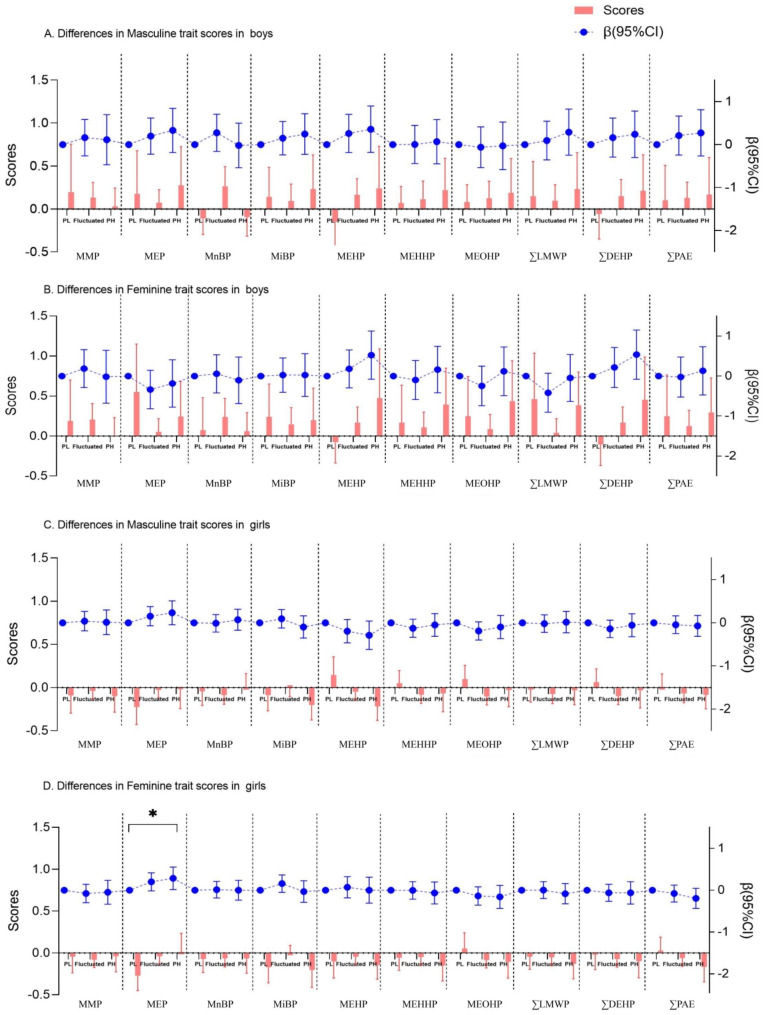
Linear associations between long-term PAEs exposure and the differences in gender trait scores between visit 1 and visit 5 in children. (*95%CI did not contain 0 referred to *P* < 0.05; PL group was considered as reference group)

**Fig. 5:**
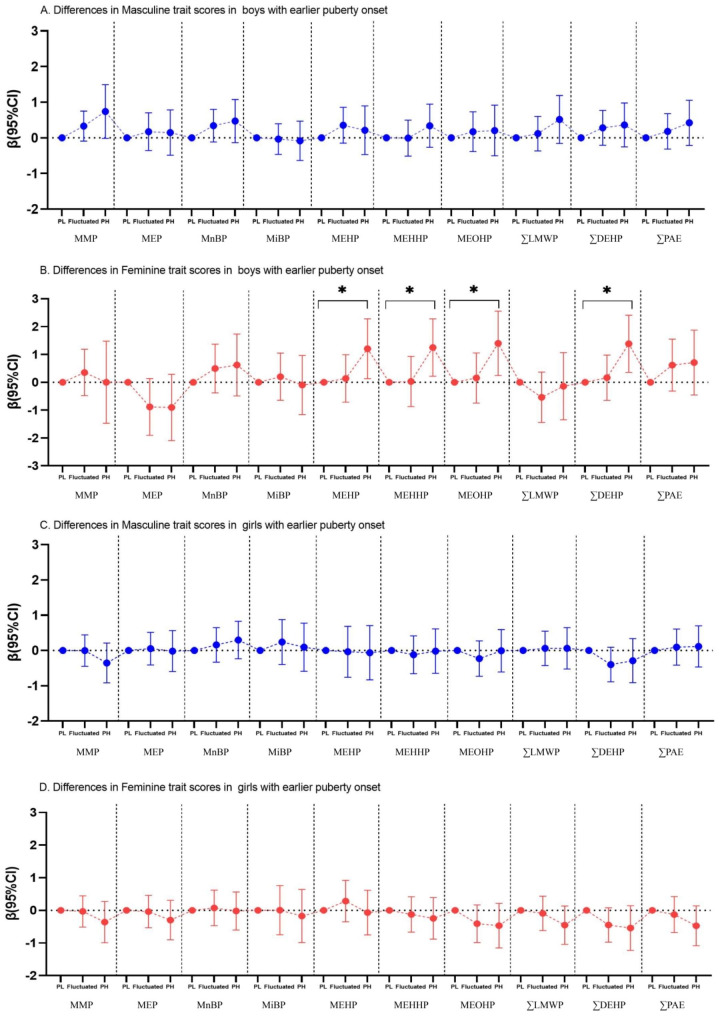
Linear associations between long-term PAEs exposure and the differences in gender trait scores between visit 1 and visit 5 in children with early pubertal onset. (*95%CI did not contain 0 referred to *P* < 0.05; PL group was considered as reference group)

In the total population, long-term exposure to PAEs might increase the femininity trait scores from visit 1 to visit 5 in boys and girls (Fig. 4), and the linear relationships between PAEs exposure and the differences of gender trait scores in boys and girls were observed (*non-linear P* > 0.05, Table S9 and Figure S7). In population with earlier pubertal onset, persistent high exposure to MEHP, MEHHP and MEOHP might elevate the femininity trait scores in boys (Figure S8). It was worth noting that persistent high exposure to MEHP (β = 1.20, 95%CI = 0.13, 2.28), MEHHP (β = 1.25, 95%CI = 0.22, 2.28) and MEOHP (β = 1.40, 95%CI = 0.24, 2.56) was significantly associated with the increased differences of femininity trait scores in boys who enter puberty earlier, prolonged exposure to DEHP might also have such a positive impact (β = 1.38, 95%CI = 0.36, 2.41) (Fig. 5). However, in the population of children who did not enter puberty earlier (Figure S9), long-term exposure to MEP might increase the masculinity and femininity trait scores in girls (*P* < 0.05). Since BMI was considered to be associated with both PAE exposure and pubertal timing, based on the adjusted model, we additionally controlled for BMI and the results remained similiar (Figure S10-S11) to the main findings.

### Phthalate exposure and gender identity

For gender identity defined in visit 5, when separated by each PAEs metabolites group, the percentages of undifferentiated type were all approximately over 40% since they were younger (Figure S12). Persistent high exposure to PAEs might be negatively related with androgyny type among children of both sexes and undifferentiated type in girls, after controlling for the gender identity in visit 1 (Table S10). In sensitivity analysis, the prevalence of four types of gender identity in children who entered puberty earlier across different PAEs exposure groups was presented in Figure S13. Persistent exposure to LMWP was negatively associated with undifferentiated type among boys entering puberty earlier (RR = 0.18, 95%CI = 0.05, 0.75, *P* < 0.05) (Table 2); and a majority of PAEs metabolites exposure showed RRs > 1 for boys’ femininity, though without statistical significance. Non significant associations between PAEs exposure and gender identity were observed among children without earlier puberty initiation (Table S11).

**Table 2:** Log-binomial analyses of relationships between log-transformed phthalates exposure and gender role in children with earlier onset of puberty

PAEs metabolites	*Boys*				*Girls*			
	**masculinity**	**femininity**	**androgyny**	**undifferentiated**	**masculinity**	**femininity**	**androgyny**	**undifferentiated**
LnMMP								
Persistent low	1.00 (reference)	1.00 (reference)	1.00 (reference)	1.00 (reference)	1.00 (reference)	1.00 (reference)	1.00 (reference)	1.00 (reference)
Fluctuated	1.03 (0.89, 1.19)	1.04 (0.13, 8.12)	1.10 (0.70, 1.74)	0.72 (0.30, 1.73)	0.65 (0.05, 8.27)	1.15 (0.81, 1.64)	1.10 (0.74, 1.63)	0.90 (0.67, 1.22)
Persistent high	1.06 (0.79, 1.42)	1.08 (0.02, 65.98)	1.22 (0.49, 3.03)	0.52 (0.09, 2.98)	0.71 (0.03, 18.04)	1.32 (0.65, 2.69)	1.21 (0.55, 2.66)	0.81 (0.44, 1.50)
LnMEP								
Persistent low	1.00 (reference)	1.00 (reference)	1.00 (reference)	1.00 (reference)	1.00 (reference)	1.00 (reference)	1.00 (reference)	1.00 (reference)
Fluctuated	1.10 (0.10, 12.36)	1.14 (0.65, 1.99)	0.90 (0.37, 2.20)	1.05 (0.46, 2.40)	1.05 (0.64, 1.74)	0.93 (0.67, 1.29)	1.06 (0.73, 1.53)	0.82 (0.44, 1.51)
Persistent high	0.62 (0.03, 13.19)	1.29 (0.42, 3.95)	0.81 (0.14, 4.84)	1.10 (0.21, 5.74)	1.10 (0.40, 3.01)	0.87 (0.46, 1.65)	1.12 (0.53, 2.34)	0.67 (0.20, 2.29)
LnMnBP								
Persistent low	1.00 (reference)	1.00 (reference)	1.00 (reference)	1.00 (reference)	1.00 (reference)	1.00 (reference)	1.00 (reference)	1.00 (reference)
Fluctuated	1.06 (0.92, 1.22)	1.01 (0.59, 1.72)	2.11 (0.77, 5.77)	0.55 (0.26, 1.18)	0.48 (0.03, 6.86)	0.91 (0.70, 1.18)	1.03 (0.72, 1.47)	0.97 (0.55, 1.73)
Persistent high	1.12 (0.84, 1.48)	1.01 (0.35, 2.96)	4.43 (0.59, 33.25)	0.30 (0.07, 1.39)	1.04 (0.07, 14.48)	0.83 (0.49, 1.39)	1.06 (0.52, 2.15)	0.95 (0.30, 2.99)
LnMiBP								
Persistent low	1.00 (reference)	1.00 (reference)	1.00 (reference)	1.00 (reference)	1.00 (reference)	1.00 (reference)	1.00 (reference)	1.00 (reference)
Fluctuated	0.97 (0.38, 2.44)	0.91 (0.51, 1.66)	0.99 (0.57, 1.70)	1.99 (0.79, 4.98)	0.29 (0.02, 3.89)	1.12 (0.82, 1.52)	0.89 (0.61, 1.29)	1.06 (0.72, 1.57)
Persistent high	0.93 (0.15, 5.94)	0.84 (0.26, 2.74)	0.97 (0.33, 2.90)	3.95 (0.63, 24.83)	0.25 (0.01, 5.62)	1.25 (0.67, 2.32)	0.79 (0.38, 1.66)	1.13 (0.52, 2.46)
LnMEHP								
Persistent low	1.00 (reference)	1.00 (reference)	1.00 (reference)	1.00 (reference)	1.00 (reference)	1.00 (reference)	1.00 (reference)	1.00 (reference)
Fluctuated	1.06 (0.55, 2.04)	0.96 (0.85, 1.08)	1.13 (0.88, 1.46)	0.88 (0.64, 1.22)	-	1.02 (0.69, 1.50)	-	1.23 (0.83, 1.84)
Persistent high	1.13 (0.30, 4.18)	0.91 (0.72, 1.16)	1.29 (0.78, 2.13)	0.78 (0.41, 1.48)	-	1.03 (0.47, 2.26)	-	1.53 (0.69, 3.39)
LnMEHHP								
Persistent low	1.00 (reference)	1.00 (reference)	1.00 (reference)	1.00 (reference)	1.00 (reference)	1.00 (reference)	1.00 (reference)	1.00 (reference)
Fluctuated	0.97 (0.69, 1.39)	1.01 (0.90, 1.12)	1.19 (0.86, 1.65)	0.47 (0.22, 1.02)	-	1.16 (0.82, 1.64)	0.92 (0.62, 1.37)	1.08 (0.73, 1.59)
Persistent high	0.95 (0.47, 1.92)	1.01 (0.81, 1.26)	1.41 (0.74, 2.71)	0.22 (0.05, 1.05)	-	1.34 (0.67, 2.68)	0.85 (0.38, 1.87)	1.16 (0.54, 2.52)
LnMEOHP								
Persistent low	1.00 (reference)	1.00 (reference)	1.00 (reference)	1.00 (reference)	1.00 (reference)	1.00 (reference)	1.00 (reference)	1.00 (reference)
Fluctuated	-	0.99 (0.87, 1.12)	-	0.70 (0.34, 1.43)	0.49 (0.04, 5.82)	0.99 (0.77, 1.28)	0.94 (0.65, 1.35)	1.09 (0.59, 2.00)
Persistent high	-	0.98 (0.76, 1.25)	-	0.48 (0.12, 2.03)	0.42 (0.02, 8.85)	0.98 (0.59, 1.65)	0.88 (0.43, 1.81)	1.19 (0.35, 4.01)
Ln∑LMWP								
Persistent low	1.00 (reference)	1.00 (reference)	1.00 (reference)	1.00 (reference)	1.00 (reference)	1.00 (reference)	1.00 (reference)	1.00 (reference)
Fluctuated	0.70 (0.10, 4.76)	1.09 (0.96, 1.24)	1.62 (0.91, 2.88)	**0.43 (0.21, 0.87)**	-	0.91 (0.71, 1.17)	0.93 (0.63, 1.37)	1.16 (0.82, 1.63)
Persistent high	0.99 (0.76, 1.30)	1.19 (0.92, 1.55)	2.62 (0.83, 8.27)	**0.18 (0.05, 0.75)**	-	0.83 (0.51, 1.37)	0.86 (0.39, 1.87)	1.34 (0.68, 2.64)
Ln∑DEHP								
Persistent low	1.00 (reference)	1.00 (reference)	1.00 (reference)	1.00 (reference)	1.00 (reference)	1.00 (reference)	1.00 (reference)	1.00 (reference)
Fluctuated	1.03 (0.72, 1.48)	1.01 (0.90, 1.13)	-	0.86 (0.38, 1.95)	1.02 (0.57, 1.86)	0.90 (0.69, 1.18)	0.92 (0.53, 1.58)	1.27 (0.75, 2.16)
Persistent high	1.07 (0.52, 2.20)	1.01 (0.80, 1.28)	-	0.74 (0.14, 3.79)	1.05 (0.32, 3.45)	0.82 (0.48, 1.39)	0.84 (0.28, 2.50)	1.62 (0.56, 4.67)
Ln∑PAEs								
Persistent low	1.00 (reference)	1.00 (reference)	1.00 (reference)	1.00 (reference)	1.00 (reference)	1.00 (reference)	1.00 (reference)	1.00 (reference)
Fluctuated	0.56 (0.08, 3.95)	1.08 (0.96, 1.22)	-	0.62 (0.32, 1.19)	-	0.93 (0.73, 1.20)	0.84 (0.55, 1.29)	1.30 (0.90, 1.87)
Persistent high	-	1.16 (0.91, 1.48)	-	0.38 (0.10, 1.42)	-	0.87 (0.53, 1.44)	0.71 (0.30, 1.67)	1.69 (0.81, 3.51)

^a^Adjusted for age, single-child status, monthly household incomes, parents’ occupation and gender identity in visit 1

^b^Bold values referred to *P* < 0.05

^c^Data were not shown if the number of participants in this group was too small to analyze

## Discussion

To date, we provided evidence that long-term PAEs exposure was significantly related with gender identity development among Chinese children. Overall, the concentration of MMP, MEP, MiBP, and MEHHP in more than 90% of participants was above the LOD values. Long-term exposure to MEHP, MEHHP and MEOHP were positively related with the increased scores of femininity trait in boys with early pubertal onset. In addition, persistent high exposure to LMWP might accelerate their gender role’s development away from undifferentiated type; and a majority of PAEs metabolites exposure showed RRs > 1 for boys’ femininity. Although the mechanisms remain to be determined, environmental pollution might have subtle, yet measurable effects on childhood gender identity.

Children are particularly vulnerable to exposure to PAEs [24], as over 80% of them are exposed to levels above LODs. Exposure to PAEs begins early in prenatal period through common sources such as widely used plastic-steel window materials, artificial leather sofas [25], perfume, hair products, shower soap and shampoo [26]. This exposure may result in a greater cumulative toxicological burden over a lifetime. Due to their hormone-like properties, the link between PAE exposure and sex-typical traits is presumed to be strong at various developmental windows. One study found that prenatal environmental contaminants may contribute to shifts in gender-role behavior [27], Specifically, masculine behavior increased among boys with age and the number of years of maternal contaminated fish consumption [27]. Moreover, gestational exposure to PAEs was associated with more oppositional and conduct problems [28], as well as a decreased (less masculine) composite score [15] only among boys. However, the latter literature obtained only a single prenatal urine sample from quite late in pregnancy, the phthalate metabolites reported may not reflect exposure during the most sensitive developmental window. Our study showed that childhood long-term exposure to MEHP, MEHHP, and MEOHP was significantly related with the increased femininity trait in boys with early pubertal onset. However, the MEHP exposure was also positively related with increased scores of masculinity trait in boys with early puberty, that revealed that a particular chemical exhibited simultaneous associations with both feminine and masculine scores in the same direction. These associations strongly suggested that femininity and masculinity should not be regarded as two distinct and opposing forms, as all individuals possess both feminine and masculine characteristics. Not to pathologize gender diversity while addressing the potential health effects of environmental chemicals is rather important.

Children who experience early puberty appear to be particularly vulnerable in the association between PAE exposure and gender identity. Puberty marks the onset of the developmental transition to adulthood through accelerated reproductive maturation and subsequent emotional and social maturation. In girls, early pubertal timing has been associated with gender dysphoria and emotional and behavioral disorders [29]. Children, especially those passing through puberty earlier, may be ill-equipped to deal with emerging sexual and other exploratory urges, such as increased dependence on alcohol [30]. Early pubertal timing could be directly related to gender identity development through early hormonal influences, or there could be a common genetic or epigenetic cause. In particular, puberty is a period when gender-related constructs, such as gender role are especially salient. Teenagers experience biological, cognitive, and social changes during puberty that can affect their gender role attitudes. In addition, sex hormones undergo significant changes from pre-puberty to post-puberty and are crucial to the development of puberty and gender role. Androgen levels are well-known predictors of sexually dimorphic behaviors in the human body [31]. For example, prenatal androgen exposure had been related to more male-like gender behaviors in childhood [32]. While the exact mechanism underlying the anti-androgenic effects of PAE exposure is still unclear, evidence links exposure to PAEs with decreased synthesis of sex steroids and interference with steroid trafficking and synthesis in the testis [33]. PAEs may also interfere with aromatase activity [34], which is responsible for converting testosterone to estradiol and plays a significant role in brain masculinization [35]. The impact of sex hormones on gender identity development is still not fully understood [36]. In addition to altering sex hormones, PAEs may disrupt brain development through other mechanisms, such as disruption of thyroid hormone signaling [37]. Studies have reported that DEHP can interfere with thyroid hormone homeostasis and lower thyroid hormone levels by activating the Ras/Akt/TRHr pathway [38].

Although these findings have not been previously reported, they do raise questions as to why boys’ gender identity appears to be more vulnerable to the plasticizers studied. Differences between boys and girls in pre- and peri-pubertal factors such as pubertal maturation and changes in arousal, motivation, and emotion may contribute to this susceptibility. In addition, ubiquitous PAEs are suspected to harm male fertility in humans. These sex differences may provide important clues for understanding the etiology of various illnesses. Recent studies have shown significant inverse associations between serum levels of PAE metabolites and serum testosterone [39], as well as reduced semen volume, total sperm count [39], and motility of spermatozoa [40] in men. These findings are consistent with the weak anti-androgenic action of PAE metabolites and may explain some of the sex-specific mechanisms observed. Furthermore, differences in the susceptibility of boys and girls to PAE exposure may be related to perceptual features of the stimuli that arouse different responses in the brain, which are governed by how the brain was organized during development by androgens [41]. However, evidence for sex differences in gender identity remains limited, and future methodological considerations are urgently needed to shed light on these complex issues.

Our results from the analyses focusing on girls were less conclusive than for boys. Among total population, exposure to MEHHP and MEOHP was negatively related with androgyny type in girls, while exposure to MEP was negatively related with their undifferentiated type. Since the binding affinity values are more negative (more stable binding) for long-chain PAEs, they are the potential disruptors of androgen and estrogen signaling [42]. What’s more, it was reported that free-testosterone and estradiol decreased with increasing ∑DEHP metabolite levels [43], the mechanisms of decreased testosterone and estradiol might explain the negative associations with androgyny type. In addition, MnBP exposure seems to be positively related with increased scores of masculinity trait onset in girls with early pubertal onset. The short-chain phthalates are common in aerosols, perfumes, creams and cosmetics widely used especially in girls, and one previous study also found that MnBP (27%) was identified as possible contributors to the mixture effect on masculine score among girls [44]. In the population of children who did not enter puberty earlier, long-term exposure to MEP might increase the masculinity and femininity trait scores in girls. Since the pubertal timing is highly polygenic, and many loci are conserved among ethnicities, epigenetic mechanisms, and several potential mechanisms, including hormone-independent ones, may be responsible for observed sex differences. PAEs are generally known to be anti-androgenic, but it may also interfere with other pathways and receptors on a molecular level, depending on the cellular and tissue context [45]. While this study primarily investigated the endocrine-related PAEs exposure, it is also crucial to recognize that gender identity arises from intricate interactions involving biological determinants as well as social and cultural influences. In addition to environmental pollutants, boys’ increased femininity traits may emerge from increased societal acceptance, legislative changes, and expanded third-party insurance coverage [46]. Besides, national and global advocacy efforts, in conjunction with transparent health care guidelines, also have resulted in opportunities for patients suffering from different gender identity opposite to their natal sex. Given the complexity of both the molecular effects of these chemicals and the biological foundations of gender identity, it is crucial to conduct further research to elucidate the relationship and the mechanisms underlying the observed associations.

Strengths of this study are apparent. To our knowledge, the longitudinal cohort design and collection of rich data, including repeated urine samples, could reflect habitual use of PAE-containing products. Also, studying school-age children provides more reliable information than that gathered from younger children because gendered patterns of behavior stabilize in the school-age years [47]. However, several limitations should be considered when interpreting these findings. Firstly, since PAEs had short biological half-lives (3–18 h) [48], urinary PAEs might only reflect exposures that occurred ≤ 1 day before the urine sample collection [49], but we have done repeated urine measurements during three consecutive years. Besides, it was possible that a follow-up duration of 3 years may not provide sufficient time to fully investigate such relationships. Second, we used the CSRI scale to assess gender identity, which might not be completely appropriate for younger pediatric population, leading to the possibility bias. However, acceptable reliability and validity of the CSRI were detected. Besides, it was possible that there were unmeasured confounders that might influence such associations, such as seafood and take-out food, which contained potential pollutants and needed to be analyzed in the further study. Thirdly, the potential role of co-exposure to other endocrine disrupting chemicals was not explored in this study but should be addressed in the future work. Fourthly, we only measured children in one coastal city in China, resulting in a limited extrapolation of exposures evaluations to other areas. Fifth, when the outcome of interest is common in the study population (> 10%), the more frequent the outcome, the more the odds ratio overestimates the risk ratio when it is more than 1 or underestimates it when it is less than 1, thus we could not deny any overestimation or underestimation of each gender identity. In summary, a paucity of studies in this area, along with sparse reporting of gender identity measurement, made it difficult to compare the relative use of tools. Besides, in the human there is a sex-dimorphism in gender identity, and the sexual dimorphic brain is considered the anatomical substrate of psychosexual development [50]. As sexual differentiation of the brain takes place later in development than sexual differentiation of the genitals, these two processes can be influenced independently by each other. Future research is required to validate existing measures and findings, and create more relevant, culturally appropriate tools.

## Conclusion

In conclusion, our findings evaluated the long-term associations between exposure to PAEs and gender identity in children. MEHP, MEHHP and MEOHP exposure were positively related with increased scores of femininity trait in boys with early pubertal onset, and exposure to LMWP might accelerate their gender role’s development away from undifferentiated type. Such results are particularly important for filling the gap of the health impact of PAEs, especially sexual health and gender identity in the critical stage of puberty development of children. Therefore, avoiding exposure to environmental endocrine compounds, strengthening industry supervision and legislation to generate similar compounds are of great public health importance.

### Electronic supplementary material

Below is the link to the electronic supplementary material.

**Supplementary File: Table S1**. Detailed parameters of PAEs metabolites detection. **Table S2**. Intraclass Correlation Coefficients for the repeated measurement data of different visits. **Table S3**. The masculine or feminine options in the Chinese version of Children’s Sex Role Inventory (CSRI) scale. **Table S4**. The results of Goodness-of-Fit test (Hosmer-Lemeshow test). **Table S5**. Characteristic of the baseline pediatric population from the longitudinal cohort study in China. **Table S6**. The concentration of phthalate metabolites and detection rate in boys and girls. **Table S7**. The concentration of phthalate metabolites and detection rate in boys and girls in three groups. **Table S8**. Approximate analysis of deviance using smoothing model analysis for the total PAEs exposure and the gender indentity scores in visit 5. **Table S9**. Approximate analysis of deviance using smoothing model analysis for the total PAEs exposure and the difference of gender indentity scores between visit 1 and visit 5. **Table S10**. Log-binomial analyses of relationships between log-transformed phthalates exposure and gender identity in visit 5 in children. **Table S11**. Log-binomial analyses of relationships between log-transformed phthalates exposure and gender identity in visit 5 in children without early onset of puberty. **Figure S1**. The appropriate scaled normal density for log-transformed values of PAEs in boys and girls. **Figure S2**. Sex-specific distribution of gender trait scores and percentage of gender identity type (* referred to P < 0.05; ** referred to P < 0.01). **Figure S3**. The results of GAM for the total PAEs exposure and the gender indentity scores in visit 5 (A. Boys; B. Girls). **Figure S4**. Distribution of gender trait scores in boys and girls with earlier pubertal onset. (*referred to significant differences). **Figure S5**. Distribution of gender trait scores in boys and girls without early pubertal onset. (*referred to significant differences). **Figure S6**. Linear associations between long-term PAEs exposure and the masculinity trait and femininity trait scores in children without early pubertal onset. (*95%CI did not contain 0 referred to P < 0.05.). **Figure S7**. The results of GAM for the total PAEs exposure and the difference of gender indentity scores between visit 1 and visit 5 (A. Boys; B. Girls). **Figure S8**. Distribution of the differences in gender trait scores between the visit 1 and the visit 5 in boys and girls with earlier pubertal onset. **Figure S9**. Distribution of the differences in gender trait scores between the visit 1 and the visit 5 and its associations with long-term PAEs exposures in boys and girls without early pubertal onset. (*95%CI did not contain 0 referred to P < 0.05.). **Figure S10**. Linear associations between long-term PAEs exposure and the differences in gender trait scores between visit 1 and visit 5 in children with early pubertal onset. (Model was additionally adjusted for BMI. *95%CI did not contain 0 referred to P <0.05.). **Figure S11**. Linear associations between long-term PAEs exposure and the differences in gender trait scores between visit 1 and visit 5 in children without early pubertal onset. (Model was additionally adjusted for BMI. *95%CI did not contain 0 referred to P < 0.05.). **Figure S12**. Distribution of gender identity in boys and girls. (Blue referred to boys and red referred to girls). **Figure S13**. Distribution of four types of gender identity in children who entered puberty earlier. (Blue referred to boys and red referred to girls).

## Data Availability

Datasets generated and/or analyzed in the present study are available from the corresponding author upon reasonable request. Proposals should be directed to harveyzou2002@bjmu.edu.cn, dongyanhui@bjmu.edu.cn.

## Abbreviations

PAEs: Phthalate esters
LMWP: Low molecular weight phthalates
HMWP: High molecular weight phthalates
DEHP: Di(2-ethylhexyl) phthalate
MnBP: Mono-n-butyl phthalate
MEHP: Mono-2-ethylhexyl phthalate
TT: Testosterone
E_2_: Estradiol
MMP: Mono-methyl phthalate
MEP: Mono-ethyl phthalate
MiBP: Mono-iso-butyl phthalate
MEHHP: Mono-2-ethyl-5-hydroxyhexyl phthalate
MEOHP: Mono-2-ethyl-5-oxohexyl phthalate
LODs: Limit of detections
CNY: Chinese Yuan
ARR: Adjusted Risk Ratios
