# Supplementary material for: Long-term exposure to exogenous phthalate, masculinity and femininity trait, and gender identity in children: a Chinese 3-year longitudinal cohort study
Source: Environ Health. 2023 Nov 28;22:81. doi: 10.1186/s12940-023-01031-5 (PMC10683128; doi:10.1186/s12940-023-01031-5)
Supplement: Supplementary file 1 — Supplementary File: Table S1. Detailed parameters of PAEs metabolites detection. Table S2. Intraclass Correlation Coefficients for the repeated measurement data of different visits. Table S3. The masculine or feminine options in the Chinese version of Children’s Sex Role Inventory (CSRI) scale. Table S4. The results of Goodness-of-Fit test (Hosmer-Lemeshow test). Table S5. Characteristic of the baseline pediatric population from the longitudinal cohort study in China. Table S6. The concentration of phthalate metabolites and detection rate in boys and girls. Table S7. The concentration of phthalate metabolites and detection rate in boys and girls in three groups. Table S8. Approximate analysis of deviance using smoothing model analysis for the total PAEs exposure and the gender indentity scores in visit 5. Table S9. Approximate analysis of deviance using smoothing model analysis for the total PAEs exposure and the difference of gender indentity scores between visit 1 and visit 5. Table S10. Log-binomial analyses of relationships between log-transformed phthalates exposure and gender identity in visit 5 in children. Table S11. Log-binomial analyses of relationships between log-transformed phthalates exposure and gender identity in visit 5 in children without early onset of puberty. Figure S1. The appropriate scaled normal density for log-transformed values of PAEs in boys and girls. Figure S2. Sex-specific distribution of gender trait scores and percentage of gender identity type (* referred to P < 0.05; ** referred to P < 0.01). Figure S3. The results of GAM for the total PAEs exposure and the gender indentity scores in visit 5 (A. Boys; B. Girls). Figure S4. Distribution of gender trait scores in boys and girls with earlier pubertal onset. (*referred to significant differences). Figure S5. Distribution of gender trait scores in boys and girls without early pubertal onset. (*referred to significant differences). Figure S6. Linear associations between long-term PAEs e [file 12940_2023_1031_MOESM1_ESM.docx]

**Supplementary File:**

Table S1. Detailed parameters of PAEs metabolites detection.

| Phthalate  metabolites | LOD  （ng/ml） | Regression equation of Standard Curve | r | Linear range of the standard curve（ng/ml） |
| --- | --- | --- | --- | --- |
| Visit 1 (May 2018) | | | | |
| MMP | 0.333 | Y=0.00735X+0.03704 | 0.99996 | 0.5~500 |
| MEP | 0.058 | Y=0.02909X+0.03518 | 0.99909 | 0.5~100 |
| MnBP | 0.368 | Y=0.00389X+0.07789 | 0.99847 | 0.5~100 |
| MiBP | 0.442 | Y=0.02913X+0.09224 | 0.99864 | 0.5~100 |
| MEHP | 0.0125 | Y=0.00988X+0.37752 | 0.99864 | 0.5~100 |
| MEHHP | 0.127 | Y=0.00033X-0.00043 | 0.99954 | 0.5~100 |
| MEOHP | 0.208 | Y=0.03100X+0.08287 | 0.99372 | 0.5~100 |
| Visit 3 and visit 5 (May 2019 and Oct 2020) | | | | |
| MMP | 0.347 | Y=0.02813X+0.03633 | 0.9992 | 0.5~100 |
| MEP | 0.500 | Y=0.01720X-0.03291 | 0.99985 | 0.5~1000 |
| MnBP | 0.120 | Y=0.00419X+0.77553 | 0.99168 | 0.5~250 |
| MiBP | 0.084 | Y=0.02432X+0.06852 | 0.99983 | 0.5~500 |
| MEHP | 0.0313 | Y=0.00991X+0.02032 | 0.99977 | 0.5~1000 |
| MEHHP | 0.0167 | Y=0.00685X-0.00338 | 0.99916 | 0.5~500 |
| MEOHP | 0.075 | Y=0.02050X-0.04089 | 0.9994 | 0.5~1000 |

Y, relative peak area ratio; X, mass concentration, ng/mL.

(Abbreviations: MMP: mono-methyl phthalate; MEP: mono-ethyl phthalate; MnBP: mono-n-butyl phthalate; MiBP: mono-iso-butyl phthalate; MEHP: mono-2-ethylhexyl phthalate; MEHHP: mono-2-ethyl-5-hydroxyhexyl phthalate; MEOHP: mono-2-ethyl-5-oxohexyl phthalate; LMWP: low molecular-weight phthalates; DEHP: di(2-ethylhexyl) phthalate; PAEs: Phthalates; LOD, Limit of Detection.)

| Table S2. Intraclass Correlation Coefficients for the repeated measurement data of different visits. | | |
| --- | --- | --- |
| ICC test | Boys | Girls |
| LnMMP | 0.234 | 0.107 |
| LnMEP | 0.363 | 0.221 |
| LnMnBP | 0.216 | 0.245 |
| LnMiBP | 0.301 | 0.321 |
| LnMEHP | 0.129 | 0.089 |
| LnMEHHP | 0.267 | 0.242 |
| LnMEOHP | 0.302 | 0.256 |
| Ln∑LMWP | 0.181 | 0.314 |
| Ln∑DEHP | 0.275 | 0.254 |
| Ln∑PAEs | 0.185 | 0.284 |

Table S3. The masculine or feminine options in the Chinese version of Children’s Sex Role Inventory (CSRI) scale .

| **Items** |
| --- |
| **Masculine options** |
| It's easier for me to make up my mind to do something |
| I can take care of myself |
| I can manage many children in our class |
| It's easy for me to express my thoughts when I have to make a decision |
| Among my friends, I am a leader |
| I like to do things my own way instead of imitating others |
| I am willing to put in effort to get what I want |
| I believe in my own abilities |
| I will persist in what I believe |
| I prefer doing things on my own rather than seeking help from others |
| I am good at sports |
| Even if I know that others may not agree, I am willing to share my views with them |
| I can leave a deep impression on most people who know me |
| Many times, I can make people do what I want them to do |
| I enjoy thinking and solving problems |
| When dealing with things, I am good at command and coordination |
| I am willing to take risks |
| **Feminine options** |
| I care about what happens to others |
| When someone is sad, I will try my best to comfort them |
| I speak very gently |
| I am a passionate person |
| I am a kind and caring person |
| I don't like to use foul language or curse people |
| I really like babies and children |
| I am a gentle person |
| I am an outgoing person |
| When I like someone, I will do something beneficial for them to express my thoughts |
| I feel very happy when others talk about my strengths |
| I am loyal to my friends |
| When others are unhappy, I also feel unhappy |
| I can sense when someone needs help |
| I am very considerate of others' difficulties |

Table S4. The results of Goodness-of-Fit test (Hosmer-Lemeshow test).

| Goodness-of-Fit test, *p-value* | masculinity | femininity | androgyny | undifferentiated |
| --- | --- | --- | --- | --- |
| **Boys** |  |  |  |  |
| LnMMP | 0.170 | 0.406 | 0.742 | 0.852 |
| LnMEP | 0.143 | 0.167 | 0.387 | 0.854 |
| LnMnBP | 0.847 | 0.242 | 0.286 | 0.169 |
| LnMiBP | 0.757 | 0.269 | 0.265 | 0.690 |
| LnMEHP | 0.396 | 0.385 | 0.232 | 0.396 |
| LnMEHHP | 0.903 | 0.504 | 0.036 | 0.948 |
| LnMEOHP | 0.373 | 0.306 | 0.293 | 0.639 |
| Ln∑LMWP | 0.214 | 0.071 | 0.568 | 0.929 |
| Ln∑DEHP | 0.757 | 0.741 | 0.114 | 0.574 |
| Ln∑PAE | 0.163 | 0.167 | 0.435 | 0.722 |
| **Girls** |  |  |  |  |
| LnMMP | 0.742 | 0.137 | 0.043 | 0.368 |
| LnMEP | 0.272 | 0.652 | 0.189 | 0.276 |
| LnMnBP | 0.081 | 0.684 | 0.242 | 0.318 |
| LnMiBP | 0.488 | 0.188 | 0.445 | 0.599 |
| LnMEHP | 0.549 | 0.076 | 0.026 | 0.190 |
| LnMEHHP | 0.083 | 0.592 | 0.048 | 0.439 |
| LnMEOHP | 0.008 | 0.541 | 0.256 | 0.463 |
| Ln∑LMWP | 0.045 | 0.746 | 0.288 | 0.635 |
| Ln∑DEHP | 0.055 | 0.201 | 0.162 | 0.265 |
| Ln∑PAE | 0.133 | 0.101 | 0.109 | 0.783 |

| Table S5. Characteristic of the baseline pediatric population from the longitudinal cohort study in China. | | | |
| --- | --- | --- | --- |
| **Baseline population (n=1430)** | **Boys (n=753)** | **Girls (n=677)** | ***P-value*** |
| **Baseline characteristic (Oct 2017)** |  |  |  |
| Age, year | 9.00±3.44 | 8.03±0.77 | <0.001 |
| Single-child status, n(%) | 215 (28.6%) | 190 (28.1%) | 0.559 |
| *Maternal occupation*^a^*, n(%)* |  |  | 0.008 |
| Civil servant | 379 (50.3%) | 328 (48.4%) |  |
| Businessman/Company employee/Worker | 218 (29.0%) | 245 (36.2%) |  |
| Housework/Unemployed/laid-off/Retirees | 145 (19.3%) | 95 (14.0%) |  |
| Missing data | 11 (1.5%) | 9 (1.3%) |  |
| *Paternal occupation*^a^*, n(%)* |  |  | 0.577 |
| Civil servant | 331 (44.0%) | 296 (43.7%) |  |
| Businessman/Company employee/Worker | 289 (38.4%) | 278 (41.1%) |  |
| Housework/Unemployed/laid-off/Retirees | 121 (16.1%) | 93 (13.7%) |  |
| Missing data | 12 (1.6%) | 10 (1.5%) |  |
| *Monthly household income, n(%)* |  |  | 0.142 |
| < 5000 CNY | 17 (2.3%) | 8 (1.2%) |  |
| 5000-12000 CNY | 97 (12.9%) | 77 (11.4%) |  |
| ≥12000 CNY | 308 (40.9%) | 310 (45.8%) |  |
| Unknown or refuse to answer | 331 (44.0%) | 282 (41.7%) |  |
| Already puberty onset, n(%) | 17 (2.3%) | 42 (6.2%) | <0.001 |
| ^a^For the employment engaged in more than one occupation, we indicated the occupation with the longest working period. CNY, Chinese yuan. | | | |

| Table S6. The concentration of phthalate metabolites and detection rate in boys and girls. | | | | | | |
| --- | --- | --- | --- | --- | --- | --- |
| **PAEs** | Visit 1 (May 2018) | | Visit 3 (May 2019) | | Visit 5 (Oct 2020) | |
|  | **Geometric Mean (95%CI), ng/ml** | **%>LOD** | **Geometric Mean (95%CI), ng/ml** | **%>LOD** | **Geometric Mean (95%CI), ng/ml** | **%>LOD** |
| **Boys** |  |  |  |  |  |  |
| MMP (ng/ml) | 31.89 (27.05-37.60) | 91.30 | 6.22 (5.61-6.90) | 98.92 | 6.03 (5.44-6.70) | 99.28 |
| MEP (ng/ml) | 8.19 (6.63-10.11) | 93.88 | 13.53 (12.20-15.02) | 100 | 15.09 (13.29-17.14) | 100 |
| MnBP (ng/ml) | 161.40 (131.40-198.40) | 94.6 | 101.70 (87.92-117.80) | 99.64 | 36.92 (25.63-53.17) | 82.01 |
| MiBP (ng/ml) | 22.96 (19.16-27.53) | 92.45 | 26.59 (24.11-29.34) | 100 | 26.08 (23.78-28.60) | 100 |
| ∑LMWP | 267.20 (222.80-320.40) | - | 177.70 (160.90-196.30) | - | 174.20 (152.60-198.80) | - |
| MEHP (ng/ml) | 0.64 (0.42-0.97) | 61.51 | 8.10 (7.51-8.74) | 100 | 9.33 (8.39-10.38) | 100 |
| MEHHP (ng/ml) | 16.42 (13.96-19.31) | 94.96 | 25.32 (23.36-27.44) | 100 | 27.60 (25.18-30.25) | 100 |
| MEOHP (ng/ml) | 2.39 (2.00-2.86) | 83.45 | 14.52 (13.55-15.56) | 100 | 16.11 (14.87-17.46) | 100 |
| ∑DEHP | 25.12 (21.49-29.37) | - | 49.25 (45.84-52.91) | - | 55.63 (51.12-60.53) | - |
| ∑PAEs | 302.00 (253.20-360.20) | - | 238.50 (219.00-259.90) | - | 250.50 (224.20-279.90) | - |
| **Girls** |  |  |  |  |  |  |
| MMP (ng/ml) | 24.63 (20.75-29.22) | 94.20 | 6.21 (5.65-6.84) | 99.66 | 6.01 (5.37-6.73) | 97.95 |
| MEP (ng/ml) | 6.98 (5.61-8.69) | 94.54 | 13.18 (11.78-14.75) | 100 | 14.87 (13.21-16.73) | 100 |
| MnBP (ng/ml) | 120.00 (98.55-146.00) | 94.54 | 84.25 (72.81-97.49) | 99.66 | 50.74 (36.35-70.83) | 84.98 |
| MiBP (ng/ml) | 15.89 (13.49-18.73) | 91.81 | 22.39 (20.48-24.48) | 100 | 21.23 (19.21-23.47) | 99.66 |
| ∑LMWP | 210.50 (177.10-250.10) | - | 156.90 (142.30-173.10) | - | 175.90 (154.50-200.30) | - |
| MEHP (ng/ml) | 0.18 (0.12-0.26) | 47.1 | 7.03 (6.53-7.57) | 100 | 9.34 (8.45-10.32) | 100 |
| MEHHP (ng/ml) | 14.40 (12.20-16.99) | 94.88 | 25.37 (23.22-27.73) | 100 | 26.37 (24.01-28.96) | 100 |
| MEOHP (ng/ml) | 1.67 (1.38-2.01) | 78.16 | 14.65 (13.61-15.77) | 100 | 15.94 (14.68-17.31) | 100 |
| ∑DEHP | 19.60 (16.83-22.84) | - | 48.28 (44.66-52.20) | - | 54.01 (49.58-58.83) | - |
| ∑PAEs | 239.50 (202.50-283.30) | - | 220.70 (203.10-239.80) | - | 251.30 (225.50-280.10) | - |
| Abbreviations: MMP, Mono-methyl phthalate; MEP, Mono-ethyl phthalate; MnBP, Mono-n-butyl phthalate; MiBP: Mono-iso-butyl phthalate; LMWP: Low molecular weight phthalates; MEHP, Mono-(2-ethyl)-hexyl phthalate; MEOHP, Mono-(2-ethyl-5-oxohexyl) phthalate; MEHHP, Mono-(2-ethyl-5-hydroxyhexyl) phthalate; DEHP, Di-(2-ethylhexyl) phthalate; PAE, phthalate; TT, testosterone; E2, estrogen. | | | | | | |
| LOD, limit of detection. | | | | | | |

| Table S7. The concentration of phthalate metabolites and detection rate in boys and girls in three groups. | | | | | | |
| --- | --- | --- | --- | --- | --- | --- |
|  | **Visit 1 (May 2018)** | | **Visit 3 (May 2019)** | | **Visit 5 (Oct 2020)** | |
|  | **Geometric Mean (95%CI), ng/ml** | **%>LOD** | **Geometric Mean (95%CI), ng/ml** | **%>LOD** | **Geometric Mean (95%CI), ng/ml** | **%>LOD** |
| **Boys** |  |  |  |  |  |  |
| MMP (ng/ml) |  |  |  |  |  |  |
| Persistent low | 14.66 (10.10-21.27) | 93.18 | 2.88 (2.22-3.73) | 93.18 | 2.98 (2.38-3.74) | 95.45 |
| Fluctuated | 30.70 (25.11-37.55) | 94.33 | 6.51 (5.81-7.29) | 100 | 6.14 (5.45-6.91) | 100 |
| Persistent high | 90.19 (74.33-109.40) | 100 | 11.66 (9.61-14.16) | 100 | 12.08 (9.99-14.62) | 100 |
| MEP (ng/ml) |  |  |  |  |  |  |
| Persistent low | 1.79 (1.12-2.87) | 86.27 | 6.72 (6.08-7.42) | 100 | 6.37 (5.74-7.06) | 100 |
| Fluctuated | 9.07 (7.06-11.66) | 96.07 | 13.08 (11.63-14.71) | 100 | 15.02 (12.92-17.46) | 100 |
| Persistent high | 27.49 (21.34-35.41) | 100 | 31.72 (24.69-40.74) | 100 | 37.72 (28.02-50.79) | 100 |
| MnBP (ng/ml) |  |  |  |  |  |  |
| Persistent low | 37.62 (19.59-72.25) | 84 | 39.37 (30.18-51.36) | 100 | 2.54 (1.00-6.42) | 54 |
| Fluctuated | 182.30 (146.10-227.40) | 96.13 | 105.20 (87.38-126.60) | 99.45 | 43.86 (28.66-67.12) | 85.08 |
| Persistent high | 476.60 (408.00-556.70) | 100 | 245.90 (211.50-285.80) | 100 | 328.20 (271.30-397.00) | 100 |
| MiBP (ng/ml) |  |  |  |  |  |  |
| Persistent low | 9.17 (6.34-13.25) | 87.5 | 12.69 (10.90-14.78) | 100 | 13.33 (11.71-15.19) | 100 |
| Fluctuated | 21.84 (16.98-28.09) | 91.36 | 26.98 (23.84-30.52) | 100 | 26.22 (23.27-29.54) | 100 |
| Persistent high | 61.96 (53.87-71.28) | 100 | 51.05 (44.66-58.35) | 100 | 48.09 (42.40-54.54) | 100 |
| MEHP (ng/ml) |  |  |  |  |  |  |
| Persistent low | 0.03 (0.02-0.06) | 23.81 | 4.91 (4.42-5.45) | 100 | 3.76 (3.15-4.50) | 100 |
| Fluctuated | 0.64 (0.39-1.05) | 61.93 | 8.19 (7.48-8.96) | 100 | 9.65 (8.60-10.82) | 100 |
| Persistent high | 16.86 (13.81-20.59) | 100 | 13.18 (11.16-15.56) | 100 | 20.97 (16.56-26.56) | 100 |
| MEHHP (ng/ml) |  |  |  |  |  |  |
| Persistent low | 7.22 (4.48-11.62) | 89.13 | 15.04 (13.63-16.59) | 100 | 14.72 (13.21-16.40) | 100 |
| Fluctuated | 15.85 (12.93-19.44) | 94.92 | 24.23 (21.95-26.74) | 100 | 26.24 (23.44-29.37) | 100 |
| Persistent high | 36.58 (33.14-40.37) | 100 | 45.13 (39.24-51.92) | 100 | 54.94 (47.76-63.21) | 100 |
| MEOHP (ng/ml) |  |  |  |  |  |  |
| Persistent low | 0.78 (0.54-1.12) | 72.5 | 9.42 (8.72-10.19) | 100 | 9.59 (8.71-10.56) | 100 |
| Fluctuated | 2.15 (1.73-2.67) | 81.28 | 13.60 (12.58-14.71) | 100 | 15.31 (13.92-16.83) | 100 |
| Persistent high | 8.59 (7.37-10.01) | 100 | 25.89 (22.51-29.78) | 100 | 29.23 (25.15-33.97) | 100 |
| ∑LMWP |  |  |  |  |  |  |
| Persistent low | 94.97 (55.98-161.10) | - | 82.63 (69.74-97.90) | - | 64.89 (49.74-84.65) | - |
| Fluctuated | 276.40 (222.50-343.40) | - | 185.20 (163.80-209.40) | - | 173.40 (149.10-201.70) | - |
| Persistent high | 663.60 (585.20-752.50) | - | 328.40 (290.60-371.10) | - | 475.10 (392.40-575.30) | - |
| ∑DEHP |  |  |  |  |  |  |
| Persistent low | 9.20 (5.73-14.76) | - | 30.71 (28.08-33.58) | - | 30.96 (27.52-34.83) | - |
| Fluctuated | 24.62 (20.52-29.53) | - | 47.55 (43.77-51.66) | - | 53.76 (48.72-59.32) | - |
| Persistent high | 64.12 (56.35-72.96) | - | 84.84 (73.01-98.59) | - | 105.20 (90.10-122.90) | - |
| ∑PAEs |  |  |  |  |  |  |
| Persistent low | 105.60 (61.94-180.00) | - | 124.00 (108.50-141.70) | - | 105.10 (86.08-128.30) | - |
| Fluctuated | 313.30 (254.30-385.90) | - | 242.80 (218.90-269.40) | - | 251.10 (220.90-285.50) | - |
| Persistent high | 722.20 (641.30-813.30) | - | 418.20 (368.60-474.50) | - | 571.10 (486.70-670.10) | - |
| **Girls** |  |  |  |  |  |  |
| MMP (ng/ml) |  |  |  |  |  |  |
| Persistent low | 9.59 (6.24-14.76) | 88.64 | 3.25 (2.77-3.82) | 100 | 2.84 (2.21-3.65) | 95.45 |
| Fluctuated | 24.34 (19.81-29.90) | 94.12 | 6.10 (5.45-6.84) | 99.51 | 6.05 (5.29-6.92) | 98.04 |
| Persistent high | 65.25 (51.56-82.57) | 100 | 12.67 (10.79-14.88) | 100 | 12.16 (10.34-14.31) | 100 |
| MEP (ng/ml) |  |  |  |  |  |  |
| Persistent low | 1.97 (1.40-2.79) | 95.56 | 5.88 (5.28-6.55) | 100 | 5.77 (5.13-6.50) | 100 |
| Fluctuated | 6.49 (4.97-8.47) | 93.03 | 13.08 (11.54-14.82) | 100 | 14.71 (12.99-16.67) | 100 |
| Persistent high | 32.09 (21.75-47.34) | 100 | 29.55 (21.59-40.44) | 100 | 38.45 (27.71-53.35) | 100 |
| MnBP (ng/ml) |  |  |  |  |  |  |
| Persistent low | 71.73 (53.33-96.48) | 96.67 | 28.76 (21.71-38.11) | 100 | 7.69 (3.41-17.33) | 68.33 |
| Fluctuated | 97.15 (72.01-131.10) | 91.81 | 85.12 (70.96-102.10) | 99.42 | 48.57 (31.49-74.92) | 85.38 |
| Persistent high | 353.00 (316.10-394.20) | 100 | 231.80 (200.00-268.60) | 100 | 355.40 (303.20-416.70) | 100 |
| MiBP (ng/ml) |  |  |  |  |  |  |
| Persistent low | 7.01 (5.04-9.76) | 90.2 | 9.89 (8.34-11.74) | 100 | 10.06 (7.81-12.96) | 100 |
| Fluctuated | 14.64 (11.83-18.11) | 89.95 | 23.30 (21.12-25.71) | 100 | 20.70 (18.62-23.02) | 100 |
| Persistent high | 46.76 (40.26-54.32) | 100 | 42.58 (37.12-48.84) | 100 | 47.73 (41.25-55.23) | 100 |
| MEHP (ng/ml) |  |  |  |  |  |  |
| Persistent low | 0.01 (0.01-0.01) | 0 | 4.01 (3.61-4.44) | 100 | 4.03 (3.51-4.64) | 100 |
| Fluctuated | 0.13 (0.08-0.20) | 42.33 | 7.01 (6.43-7.64) | 100 | 9.00 (8.04-10.07) | 100 |
| Persistent high | 6.23 (4.18-9.29) | 100 | 10.33 (8.82-12.10) | 100 | 19.22 (15.86-23.29) | 100 |
| MEHHP (ng/ml) |  |  |  |  |  |  |
| Persistent low | 7.51 (5.41-10.41) | 94.23 | 13.05 (11.68-14.57) | 100 | 12.57 (10.66-14.84) | 100 |
| Fluctuated | 13.24 (10.67-16.44) | 93.68 | 26.00 (23.35-28.96) | 100 | 26.82 (24.05-29.90) | 100 |
| Persistent high | 38.14 (30.44-47.80) | 100 | 45.63 (38.41-54.22) | 100 | 52.71 (45.59-60.93) | 100 |
| MEOHP (ng/ml) |  |  |  |  |  |  |
| Persistent low | 0.39 (0.29-0.53) | 52.08 | 8.66 (7.95-9.44) | 100 | 8.57 (7.62-9.65) | 100 |
| Fluctuated | 1.63 (1.31-2.03) | 78.97 | 14.69 (13.47-16.02) | 100 | 15.76 (14.31-17.35) | 100 |
| Persistent high | 7.38 (5.95-9.17) | 100 | 23.98 (20.18-28.50) | 100 | 30.24 (26.22-34.89) | 100 |
| ∑LMWP |  |  |  |  |  |  |
| Persistent low | 124.20 (94.86-162.50) | - | 70.43 (59.86-82.86) | - | 66.91 (54.22-82.57) | - |
| Fluctuated | 183.30 (142.10-236.40) | - | 161.30 (143.80-181.00) | - | 171.80 (146.60-201.40) | - |
| Persistent high | 534.00 (469.10-607.90) | - | 317.70 (274.50-367.70) | - | 488.30 (419.10-569.10) | - |
| ∑DEHP |  |  |  |  |  |  |
| Persistent low | 9.20 (6.83-12.38) | - | 27.86 (25.56-30.37) | - | 29.34 (26.23-32.83) | - |
| Fluctuated | 18.72 (15.48-22.64) | - | 49.27 (44.91-54.06) | - | 54.60 (49.28-60.49) | - |
| Persistent high | 55.95 (44.00-71.14) | - | 83.66 (69.83-100.20) | - | 104.10 (88.72-122.00) | - |
| ∑PAEs |  |  |  |  |  |  |
| Persistent low | 137.20 (100.40-187.70) | - | 120.00 (107.50-133.80) | - | 109.00 (93.11-127.70) | - |
| Fluctuated | 208.00 (163.00-265.40) | - | 216.00 (195.00-239.20) | - | 246.20 (215.50-281.30) | - |
| Persistent high | 598.10 (527.80-677.90) | - | 414.60 (363.60-472.60) | - | 581.60 (505.70-668.80) | - |

Table S8. Approximate analysis of deviance using smoothing model analysis for the total PAEs exposure and the gender indentity scores in visit 5.

| GAM model, *p-value** | Boys | | Girls | |
| --- | --- | --- | --- | --- |
|  | *Masculine trait scores* | *Feminine trait scores* | *Masculine trait scores* | *Feminine trait scores* |
| **Visit 1** |  |  |  |  |
| LnMMP | 0.011 | 0.038 | 0.330 | 0.566 |
| LnMEP | 0.136 | 0.272 | 0.246 | 0.228 |
| LnMnBP | 0.054 | 0.213 | 0.160 | 0.401 |
| LnMiBP | 0.002 | 0.003 | 0.004 | 0.023 |
| LnMEHP | 0.817 | 0.762 | 0.231 | 0.517 |
| LnMEHHP | 0.255 | 0.431 | 0.088 | 0.067 |
| LnMEOHP | 0.728 | 0.923 | 0.454 | 0.623 |
| LnLMWP | 0.044 | 0.085 | 0.206 | 0.462 |
| LnDEHP | 0.547 | 0.657 | 0.110 | 0.183 |
| LnPAE | 0.405 | 0.085 | 0.152 | 0.369 |
| **Visit 3** |  |  |  |  |
| LnMMP | 0.288 | 0.279 | 0.106 | 0.185 |
| LnMEP | 0.757 | 0.494 | 0.488 | 0.698 |
| LnMnBP | 0.633 | 0.328 | 0.388 | 0.683 |
| LnMiBP | 0.456 | 0.456 | 0.317 | 0.564 |
| LnMEHP | 0.112 | 0.056 | 0.655 | 0.462 |
| LnMEHHP | 0.687 | 0.757 | 0.719 | 0.668 |
| LnMEOHP | 0.647 | 0.666 | 0.838 | 0.719 |
| LnLMWP | 0.149 | 0.048 | 0.309 | 0.361 |
| LnDEHP | 0.648 | 0.689 | 0.573 | 0.685 |
| LnPAE | 0.125 | 0.023 | 0.226 | 0.509 |
| **Visit 5** |  |  |  |  |
| LnMMP | 0.653 | 0.602 | 0.446 | 0.144 |
| LnMEP | 0.699 | 0.333 | 0.377 | 0.095 |
| LnMnBP | 0.536 | 0.757 | 0.217 | 0.055 |
| LnMiBP | 0.300 | 0.399 | 0.598 | 0.142 |
| LnMEHP | 0.469 | 0.429 | 0.438 | 0.865 |
| LnMEHHP | 0.496 | 0.605 | 0.286 | 0.356 |
| LnMEOHP | 0.449 | 0.325 | 0.540 | 0.363 |
| LnLMWP | 0.764 | 0.788 | 0.321 | 0.063 |
| LnDEHP | 0.563 | 0.525 | 0.358 | 0.378 |
| LnPAE | 0.781 | 0.932 | 0.384 | 0.085 |

*The results of approximate analysis of deviance using smoothing model analysis in GAM model.

Table S9. Approximate analysis of deviance using smoothing model analysis for the total PAEs exposure and the difference of gender indentity scores between visit 1 and visit 5.

| GAM model, *p-value** | Boys | | Girls | |
| --- | --- | --- | --- | --- |
|  | *Masculine trait scores* | *Feminine trait scores* | *Masculine trait scores* | *Feminine trait scores* |
| **Visit 1** |  |  |  |  |
| LnMMP | 0.236 | 0.701 | 0.366 | 0.317 |
| LnMEP | 0.105 | 0.115 | 0.554 | 0.366 |
| LnMnBP | 0.070 | 0.197 | 0.063 | 0.184 |
| LnMiBP | 0.115 | 0.020 | 0.745 | 0.481 |
| LnMEHP | 0.619 | 0.526 | 0.170 | 0.776 |
| LnMEHHP | 0.639 | 0.761 | 0.028 | 0.023 |
| LnMEOHP | 0.738 | 0.892 | 0.034 | 0.084 |
| LnLMWP | 0.044 | 0.082 | 0.010 | 0.234 |
| LnDEHP | 0.619 | 0.512 | 0.343 | 0.032 |
| LnPAE | 0.039 | 0.113 | 0.362 | 0.343 |
| **Visit 3** |  |  |  |  |
| LnMMP | 0.653 | 0.831 | 0.597 | 0.732 |
| LnMEP | 0.778 | 0.514 | 0.765 | 0.762 |
| LnMnBP | 0.357 | 0.450 | 0.491 | 0.614 |
| LnMiBP | 0.259 | 0.389 | 0.099 | 0.463 |
| LnMEHP | 0.188 | 0.105 | 0.229 | 0.163 |
| LnMEHHP | 0.709 | 0.607 | 0.163 | 0.484 |
| LnMEOHP | 0.136 | 0.177 | 0.202 | 0.336 |
| LnLMWP | 0.360 | 0.029 | 0.549 | 0.263 |
| LnDEHP | 0.132 | 0.273 | 0.097 | 0.297 |
| LnPAE | 0.276 | 0.032 | 0.398 | 0.311 |
| **Visit 5** |  |  |  |  |
| LnMMP | 0.531 | 0.747 | 0.615 | 0.489 |
| LnMEP | 0.749 | 0.608 | 0.176 | 0.019 |
| LnMnBP | 0.686 | 0.751 | 0.297 | 0.129 |
| LnMiBP | 0.645 | 0.364 | 0.728 | 0.292 |
| LnMEHP | 0.140 | 0.354 | 0.278 | 0.621 |
| LnMEHHP | 0.765 | 0.682 | 0.748 | 0.734 |
| LnMEOHP | 0.728 | 0.693 | 0.839 | 0.798 |
| LnLMWP | 0.212 | 0.774 | 0.355 | 0.045 |
| LnDEHP | 0.618 | 0.658 | 0.500 | 0.712 |
| LnPAE | 0.302 | 0.861 | 0.389 | 0.109 |

*The results of approximate analysis of deviance using smoothing model analysis in GAM model.

| Table S10. Log-binomial analyses of relationships between log-transformed phthalates exposure and gender identity in visit 5 in children. | | | | | | | | |
| --- | --- | --- | --- | --- | --- | --- | --- | --- |
| PAEs metabolites | *Boys* | | | | *Girls* | | | |
|  | **masculinity** | **femininity** | **androgyny** | **undifferentiated** | **masculinity** | **femininity** | **androgyny** | **undifferentiated** |
| LnMMP |  |  |  |  |  |  |  |  |
| Persistent low | 1.00 (reference) | 1.00 (reference) | 1.00 (reference) | 1.00 (reference) | 1.00 (reference) | 1.00 (reference) | 1.00 (reference) | 1.00 (reference) |
| Fluctuated | 1.07 (0.92, 1.25) | 1.10 (0.95, 1.26) | 1.01 (0.83, 1.24) | 0.80 (0.58, 1.10) | 1.00 (0.88, 1.14) | 1.04 (0.90, 1.21) | 1.11 (0.86, 1.42) | 0.81 (0.60, 1.09) |
| Persistent high | 1.14 (0.84, 1.55) | 1.20 (0.90, 1.60) | 1.03 (0.68, 1.54) | 0.63 (0.33, 1.21) | 1.00 (0.77, 1.30) | 1.08 (0.80, 1.46) | 1.23 (0.75, 2.02) | 0.65 (0.36, 1.20) |
| LnMEP |  |  |  |  |  |  |  |  |
| Persistent low | 1.00 (reference) | 1.00 (reference) | 1.00 (reference) | 1.00 (reference) | 1.00 (reference) | 1.00 (reference) | 1.00 (reference) | 1.00 (reference) |
| Fluctuated | 1.07 (0.93, 1.22) | 1.08 (0.95, 1.24) | **0.83 (0.70, 0.98)** | 1.07 (0.75, 1.52) | 1.01 (0.89, 1.14) | 1.07 (0.94, 1.22) | 1.05 (0.83, 1.32) | **0.72 (0.54, 0.96)** |
| Persistent high | 1.14 (0.86, 1.49) | 1.17 (0.90, 1.53) | **0.69 (0.49, 0.96)** | 1.14 (0.56, 2.30) | 1.02 (0.79, 1.30) | 1.15 (0.89, 1.49) | 1.10 (0.69, 1.74) | **0.53 (0.30, 0.93)** |
| LnMnBP |  |  |  |  |  |  |  |  |
| Persistent low | 1.00 (reference) | 1.00 (reference) | 1.00 (reference) | 1.00 (reference) | 1.00 (reference) | 1.00 (reference) | 1.00 (reference) | 1.00 (reference) |
| Fluctuated | 1.11 (0.96, 1.28) | 1.06 (0.93, 1.20) | 0.96 (0.81, 1.15) | 0.80 (0.58, 1.10) | 1.04 (0.93, 1.16) | 0.99 (0.88, 1.11) | 1.08 (0.88, 1.31) | 0.90 (0.67, 1.20) |
| Persistent high | 1.22 (0.91, 1.64) | 1.11 (0.86, 1.44) | 0.93 (0.65, 1.32) | 0.64 (0.34, 1.22) | 1.08 (0.86, 1.36) | 0.98 (0.77, 1.24) | 1.16 (0.78, 1.73) | 0.81 (0.45, 1.44) |
| LnMiBP |  |  |  |  |  |  |  |  |
| Persistent low | 1.00 (reference) | 1.00 (reference) | 1.00 (reference) | 1.00 (reference) | 1.00 (reference) | 1.00 (reference) | 1.00 (reference) | 1.00 (reference) |
| Fluctuated | 1.03 (0.92, 1.14) | 0.97 (0.88, 1.08) | 0.94 (0.79, 1.13) | 1.16 (0.81, 1.65) | 0.94 (0.80, 1.09) | 1.01 (0.88, 1.16) | 0.89 (0.74, 1.08) | 1.19 (0.84, 1.70) |
| Persistent high | 1.05 (0.85, 1.31) | 0.94 (0.77, 1.16) | 0.89 (0.62, 1.27) | 1.34 (0.66, 2.71) | 0.88 (0.64, 1.20) | 1.03 (0.78, 1.35) | 0.80 (0.55, 1.16) | 1.42 (0.70, 2.88) |
| LnMEHP |  |  |  |  |  |  |  |  |
| Persistent low | 1.00 (reference) | 1.00 (reference) | 1.00 (reference) | 1.00 (reference) | 1.00 (reference) | 1.00 (reference) | 1.00 (reference) | 1.00 (reference) |
| Fluctuated | 0.98 (0.85, 1.12) | 0.93 (0.82, 1.05) | 1.15 (0.89, 1.48) | 0.96 (0.69, 1.34) | 1.01 (0.89, 1.16) | 1.01 (0.88, 1.17) | 0.82 (0.68, 1.01) | 1.17 (0.81, 1.68) |
| Persistent high | 0.95 (0.72, 1.26) | 0.87 (0.68, 1.11) | 1.32 (0.80, 2.19) | 0.92 (0.47, 1.80) | 1.03 (0.79, 1.34) | 1.03 (0.78, 1.36) | 0.68 (0.46, 1.01) | 1.36 (0.66, 2.81) |
| LnMEHHP |  |  |  |  |  |  |  |  |
| Persistent low | 1.00 (reference) | 1.00 (reference) | 1.00 (reference) | 1.00 (reference) | 1.00 (reference) | 1.00 (reference) | 1.00 (reference) | 1.00 (reference) |
| Fluctuated | 1.00 (0.87, 1.15) | 1.00 (0.89, 1.12) | 1.13 (0.92, 1.38) | 0.82 (0.61, 1.10) | 1.07 (0.94, 1.21) | 1.04 (0.92, 1.17) | **0.83 (0.70, 0.98)** | 1.10 (0.78, 1.55) |
| Persistent high | 1.00 (0.75, 1.33) | 1.00 (0.80, 1.26) | 1.27 (0.85, 1.89) | 0.67 (0.37, 1.20) | 1.14 (0.88, 1.46) | 1.08 (0.85, 1.38) | **0.69 (0.49, 0.97)** | 1.22 (0.61, 2.42) |
| LnMEOHP |  |  |  |  |  |  |  |  |
| Persistent low | 1.00 (reference) | 1.00 (reference) | 1.00 (reference) | 1.00 (reference) | 1.00 (reference) | 1.00 (reference) | 1.00 (reference) | 1.00 (reference) |
| Fluctuated | 0.99 (0.88, 1.11) | 0.98 (0.87, 1.11) | 1.05 (0.87, 1.28) | 1.03 (0.75, 1.41) | 1.07 (0.94, 1.23) | 1.01 (0.89, 1.15) | **0.79 (0.67, 0.93)** | 1.26 (0.87, 1.82) |
| Persistent high | 0.98 (0.78, 1.24) | 0.97 (0.76, 1.23) | 1.11 (0.75, 1.63) | 1.06 (0.56, 1.98) | 1.15 (0.88, 1.51) | 1.02 (0.79, 1.32) | **0.63 (0.45, 0.86)** | 1.58 (0.75, 3.30) |
| Ln∑LMWP |  |  |  |  |  |  |  |  |
| Persistent low | 1.00 (reference) | 1.00 (reference) | 1.00 (reference) | 1.00 (reference) | 1.00 (reference) | 1.00 (reference) | 1.00 (reference) | 1.00 (reference) |
| Fluctuated | 1.05 (0.91, 1.20) | 1.09 (0.95, 1.24) | 0.94 (0.80, 1.10) | 0.91 (0.65, 1.29) | 0.99 (0.89, 1.11) | 0.99 (0.87, 1.13) | 1.03 (0.85, 1.25) | 1.02 (0.75, 1.39) |
| Persistent high | 1.10 (0.83, 1.45) | 1.19 (0.91, 1.55) | 0.88 (0.64, 1.21) | 0.83 (0.42, 1.65) | 0.99 (0.79, 1.23) | 0.97 (0.75, 1.27) | 1.06 (0.71, 1.57) | 1.04 (0.56, 1.93) |
| Ln∑DEHP |  |  |  |  |  |  |  |  |
| Persistent low | 1.00 (reference) | 1.00 (reference) | 1.00 (reference) | 1.00 (reference) | 1.00 (reference) | 1.00 (reference) | 1.00 (reference) | 1.00 (reference) |
| Fluctuated | 0.96 (0.84, 1.10) | 0.99 (0.87, 1.11) | 1.05 (0.86, 1.28) | 1.06 (0.76, 1.49) | 1.03 (0.91, 1.18) | 1.00 (0.87, 1.15) | **0.81 (0.69, 0.96)** | 1.41 (0.94, 2.12) |
| Persistent high | 0.92 (0.71, 1.20) | 0.97 (0.76, 1.24) | 1.10 (0.74, 1.63) | 1.12 (0.57, 2.21) | 1.06 (0.82, 1.38) | 1.00 (0.76, 1.32) | **0.66 (0.48, 0.92)** | 1.98 (0.88, 4.48) |
| Ln∑PAEs |  |  |  |  |  |  |  |  |
| Persistent low | 1.00 (reference) | 1.00 (reference) | 1.00 (reference) | 1.00 (reference) | 1.00 (reference) | 1.00 (reference) | 1.00 (reference) | 1.00 (reference) |
| Fluctuated | 1.06 (0.93, 1.21) | 1.05 (0.93, 1.19) | 0.92 (0.78, 1.08) | 0.95 (0.67, 1.33) | 0.98 (0.88, 1.09) | 1.01 (0.90, 1.13) | 0.93 (0.78, 1.10) | 1.15 (0.84, 1.59) |
| Persistent high | 1.12 (0.86, 1.46) | 1.11 (0.87, 1.42) | 0.84 (0.61, 1.16) | 0.90 (0.45, 1.78) | 0.96 (0.78, 1.19) | 1.02 (0.82, 1.28) | 0.86 (0.61, 1.22) | 1.33 (0.70, 2.52) |
| Adjusted for age, single-child status, monthly household incomes, parents' occupation and gender identity in visit 1. Bold values referred to *P*<0.05. | | | | | | | | |

| Table S11. Log-binomial analyses of relationships between log-transformed phthalates exposure and gender identity in visit 5 in children without early onset of puberty. | | | | | | | | |
| --- | --- | --- | --- | --- | --- | --- | --- | --- |
| PAEs metabolites | *Boys* | | | | *Girls* | | | |
|  | **masculinity** | **femininity** | **androgyny** | **undifferentiated** | **masculinity** | **femininity** | **androgyny** | **undifferentiated** |
| LnMMP |  |  |  |  |  |  |  |  |
| Persistent low | 1.00 (reference) | 1.00 (reference) | 1.00 (reference) | 1.00 (reference) | 1.00 (reference) | 1.00 (reference) | 1.00 (reference) | 1.00 (reference) |
| Fluctuated | 1.11 (0.93, 1.32) | 1.09 (0.93, 1.28) | 0.98 (0.78, 1.23) | 0.78 (0.53, 1.14) | 1.00 (0.89, 1.13) | 1.04 (0.88, 1.24) | 1.13 (0.78, 1.62) | 0.94 (0.71, 1.26) |
| Persistent high | 1.23 (0.86, 1.74) | 1.19 (0.86, 1.65) | 0.95 (0.60, 1.51) | 0.61 (0.29, 1.30) | 1.00 (0.79, 1.28) | 1.09 (0.77, 1.54) | 1.27 (0.61, 2.62) | 0.89 (0.51, 1.58) |
| LnMEP |  |  |  |  |  |  |  |  |
| Persistent low | 1.00 (reference) | 1.00 (reference) | 1.00 (reference) | 1.00 (reference) | 1.00 (reference) | 1.00 (reference) | 1.00 (reference) | 1.00 (reference) |
| Fluctuated | 1.12 (0.94, 1.34) | 1.01 (0.87, 1.18) | 0.86 (0.73, 1.03) | 1.15 (0.78, 1.70) | 0.99 (0.88, 1.12) | 1.22 (0.98, 1.52) | 1.12 (0.80, 1.56) | 0.75 (0.58, 0.97) |
| Persistent high | 1.26 (0.88, 1.81) | 1.02 (0.75, 1.38) | 0.75 (0.53, 1.06) | 1.33 (0.61, 2.89) | 0.98 (0.78, 1.24) | 1.49 (0.96, 2.31) | 1.25 (0.64, 2.45) | 0.56 (0.33, 0.94) |
| LnMnBP |  |  |  |  |  |  |  |  |
| Persistent low | 1.00 (reference) | 1.00 (reference) | 1.00 (reference) | 1.00 (reference) | 1.00 (reference) | 1.00 (reference) | 1.00 (reference) | 1.00 (reference) |
| Fluctuated | 1.16 (0.97, 1.38) | 1.07 (0.93, 1.24) | 0.87 (0.74, 1.03) | 0.93 (0.64, 1.37) | 0.96 (0.79, 1.18) | 1.08 (0.90, 1.30) | 1.30 (0.93, 1.81) | 0.88 (0.67, 1.15) |
| Persistent high | 1.34 (0.94, 1.90) | 1.15 (0.86, 1.54) | 0.76 (0.54, 1.05) | 0.87 (0.40, 1.87) | 0.93 (0.62, 1.40) | 1.17 (0.81, 1.70) | 1.69 (0.87, 3.29) | 0.77 (0.44, 1.33) |
| LnMiBP |  |  |  |  |  |  |  |  |
| Persistent low | 1.00 (reference) | 1.00 (reference) | 1.00 (reference) | 1.00 (reference) | 1.00 (reference) | 1.00 (reference) | 1.00 (reference) | 1.00 (reference) |
| Fluctuated | 1.06 (0.91, 1.22) | 1.00 (0.88, 1.13) | 0.95 (0.77, 1.17) | 1.05 (0.73, 1.51) | 0.99 (0.84, 1.17) | 0.89 (0.75, 1.05) | 0.92 (0.67, 1.25) | 1.24 (0.86, 1.77) |
| Persistent high | 1.12 (0.84, 1.49) | 0.99 (0.77, 1.27) | 0.91 (0.60, 1.38) | 1.10 (0.53, 2.28) | 0.98 (0.70, 1.38) | 0.79 (0.56, 1.10) | 0.84 (0.45, 1.56) | 1.53 (0.74, 3.14) |
| LnMEHP |  |  |  |  |  |  |  |  |
| Persistent low | 1.00 (reference) | 1.00 (reference) | 1.00 (reference) | 1.00 (reference) | 1.00 (reference) | 1.00 (reference) | 1.00 (reference) | 1.00 (reference) |
| Fluctuated | 0.96 (0.83, 1.10) | 0.93 (0.81, 1.07) | 0.98 (0.75, 1.26) | 1.14 (0.76, 1.72) | 0.97 (0.80, 1.19) | 1.11 (0.88, 1.39) | 0.95 (0.67, 1.35) | 0.94 (0.70, 1.27) |
| Persistent high | 0.91 (0.69, 1.21) | 0.87 (0.66, 1.15) | 0.95 (0.57, 1.59) | 1.30 (0.57, 2.96) | 0.95 (0.64, 1.41) | 1.23 (0.78, 1.94) | 0.90 (0.44, 1.83) | 0.89 (0.49, 1.61) |
| LnMEHHP |  |  |  |  |  |  |  |  |
| Persistent low | 1.00 (reference) | 1.00 (reference) | 1.00 (reference) | 1.00 (reference) | 1.00 (reference) | 1.00 (reference) | 1.00 (reference) | 1.00 (reference) |
| Fluctuated | 1.01 (0.85, 1.22) | 1.00 (0.88, 1.14) | 1.08 (0.86, 1.35) | 0.87 (0.63, 1.20) | 1.02 (0.92, 1.14) | 0.96 (0.80, 1.14) | 0.85 (0.64, 1.13) | 1.13 (0.80, 1.58) |
| Persistent high | 1.03 (0.72, 1.48) | 1.00 (0.77, 1.30) | 1.16 (0.74, 1.81) | 0.76 (0.40, 1.45) | 1.05 (0.84, 1.30) | 0.91 (0.64, 1.30) | 0.73 (0.41, 1.29) | 1.27 (0.64, 2.49) |
| LnMEOHP |  |  |  |  |  |  |  |  |
| Persistent low | 1.00 (reference) | 1.00 (reference) | 1.00 (reference) | 1.00 (reference) | 1.00 (reference) | 1.00 (reference) | 1.00 (reference) | 1.00 (reference) |
| Fluctuated | 0.97 (0.83, 1.13) | 0.99 (0.87, 1.12) | 1.03 (0.82, 1.28) | 1.12 (0.78, 1.61) | 1.02 (0.91, 1.14) | 0.96 (0.80, 1.15) | 0.74 (0.58, 0.96) | 1.26 (0.87, 1.83) |
| Persistent high | 0.93 (0.69, 1.27) | 0.97 (0.76, 1.25) | 1.06 (0.68, 1.64) | 1.26 (0.61, 2.58) | 1.04 (0.83, 1.31) | 0.92 (0.64, 1.32) | 0.55 (0.33, 0.92) | 1.59 (0.76, 3.35) |
| Ln∑LMWP |  |  |  |  |  |  |  |  |
| Persistent low | 1.00 (reference) | 1.00 (reference) | 1.00 (reference) | 1.00 (reference) | 1.00 (reference) | 1.00 (reference) | 1.00 (reference) | 1.00 (reference) |
| Fluctuated | 1.08 (0.91, 1.27) | 1.11 (0.95, 1.29) | 0.88 (0.74, 1.05) | 1.08 (0.72, 1.62) | - | 1.02 (0.86, 1.21) | 1.31 (0.94, 1.83) | 0.92 (0.69, 1.22) |
| Persistent high | 1.16 (0.84, 1.61) | 1.23 (0.90, 1.67) | 0.78 (0.55, 1.10) | 1.16 (0.51, 2.62) | - | 1.05 (0.75, 1.46) | 1.72 (0.88, 3.36) | 0.84 (0.48, 1.48) |
| Ln∑DEHP |  |  |  |  |  |  |  |  |
| Persistent low | 1.00 (reference) | 1.00 (reference) | 1.00 (reference) | 1.00 (reference) | 1.00 (reference) | 1.00 (reference) | 1.00 (reference) | 1.00 (reference) |
| Fluctuated | - | 0.97 (0.85, 1.11) | 1.08 (0.85, 1.38) | 1.06 (0.73, 1.54) | 0.97 (0.78, 1.20) | 1.04 (0.87, 1.23) | 0.81 (0.62, 1.05) | 1.22 (0.85, 1.74) |
| Persistent high | - | 0.95 (0.72, 1.24) | 1.17 (0.73, 1.89) | 1.13 (0.53, 2.39) | 0.94 (0.61, 1.44) | 1.07 (0.77, 1.51) | 0.65 (0.38, 1.11) | 1.48 (0.73, 3.03) |
| Ln∑PAEs |  |  |  |  |  |  |  |  |
| Persistent low | 1.00 (reference) | 1.00 (reference) | 1.00 (reference) | 1.00 (reference) | 1.00 (reference) | 1.00 (reference) | 1.00 (reference) | 1.00 (reference) |
| Fluctuated | 1.11 (0.94, 1.30) | 1.08 (0.94, 1.24) | 0.88 (0.74, 1.04) | 1.09 (0.73, 1.62) | - | 1.05 (0.88, 1.26) | 1.11 (0.83, 1.48) | 0.98 (0.74, 1.30) |
| Persistent high | 1.22 (0.89, 1.68) | 1.16 (0.88, 1.55) | 0.77 (0.54, 1.09) | 1.18 (0.53, 2.63) | - | 1.11 (0.78, 1.59) | 1.22 (0.68, 2.19) | 0.97 (0.55, 1.68) |
| ^a^Adjusted for age, single-child status, monthly household incomes, parents' occupation and gender identity in visit 1.  ^b^Bold values referred to *P*<0.05.  ^c^Data were not shown if the number of participants in this group was too small to analyze. | | | | | | | | |


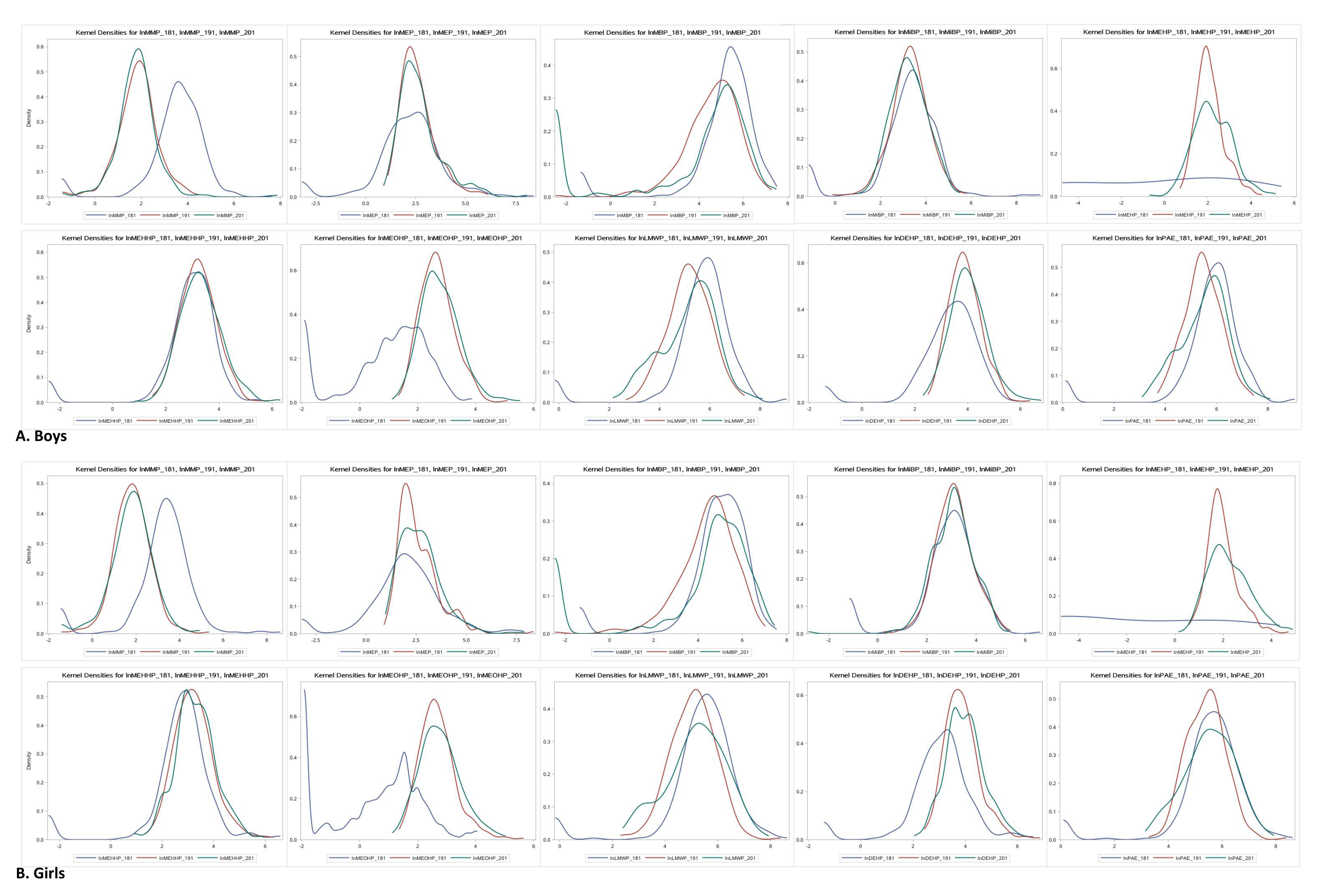


Figure S1. The appropriate scaled normal density for log-transformed values of PAEs in boys and girls.


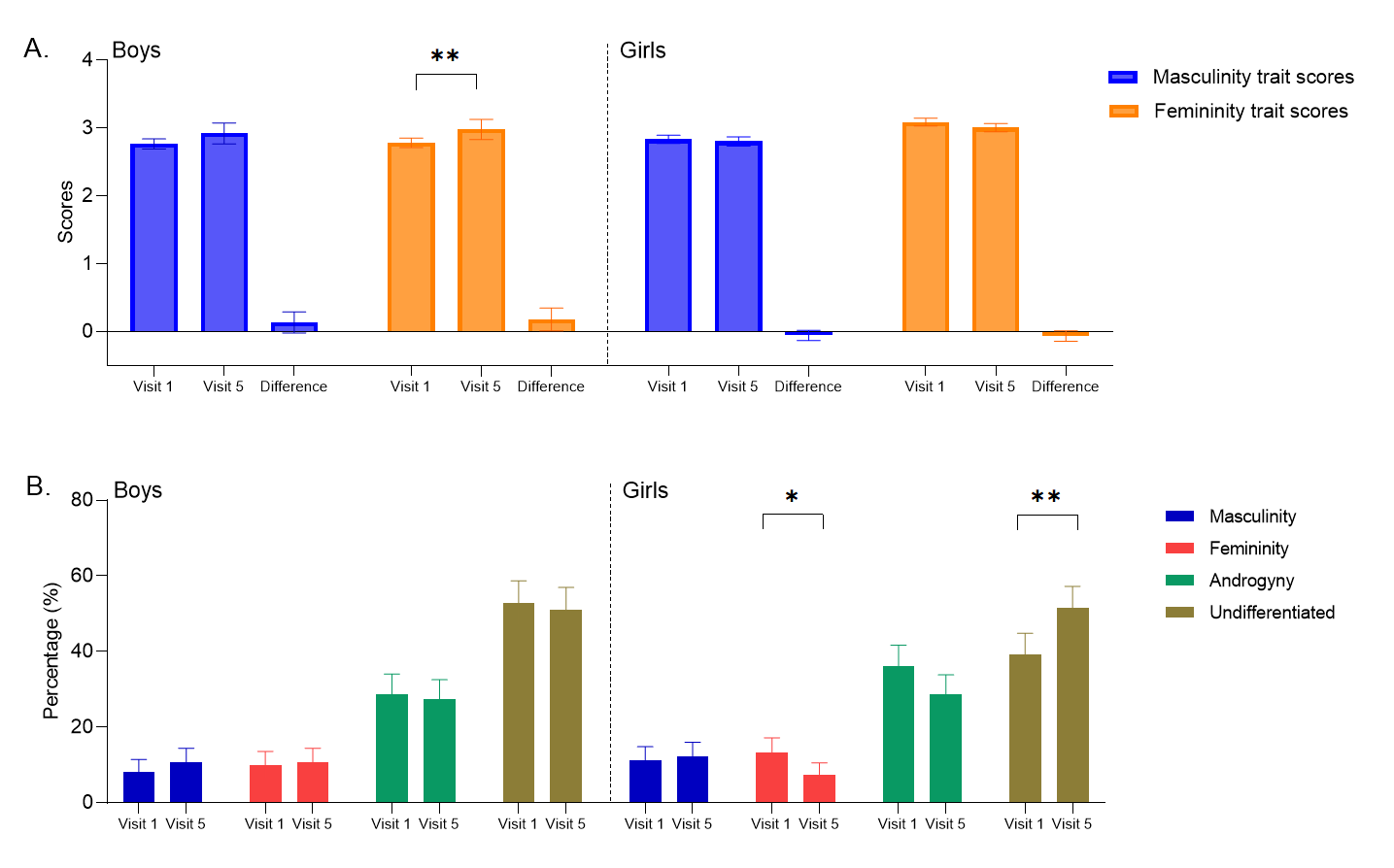


Figure S2. Sex-specific distribution of gender trait scores and percentage of gender identity type (* referred to *P*<0.05; ** referred to *P*<0.01).


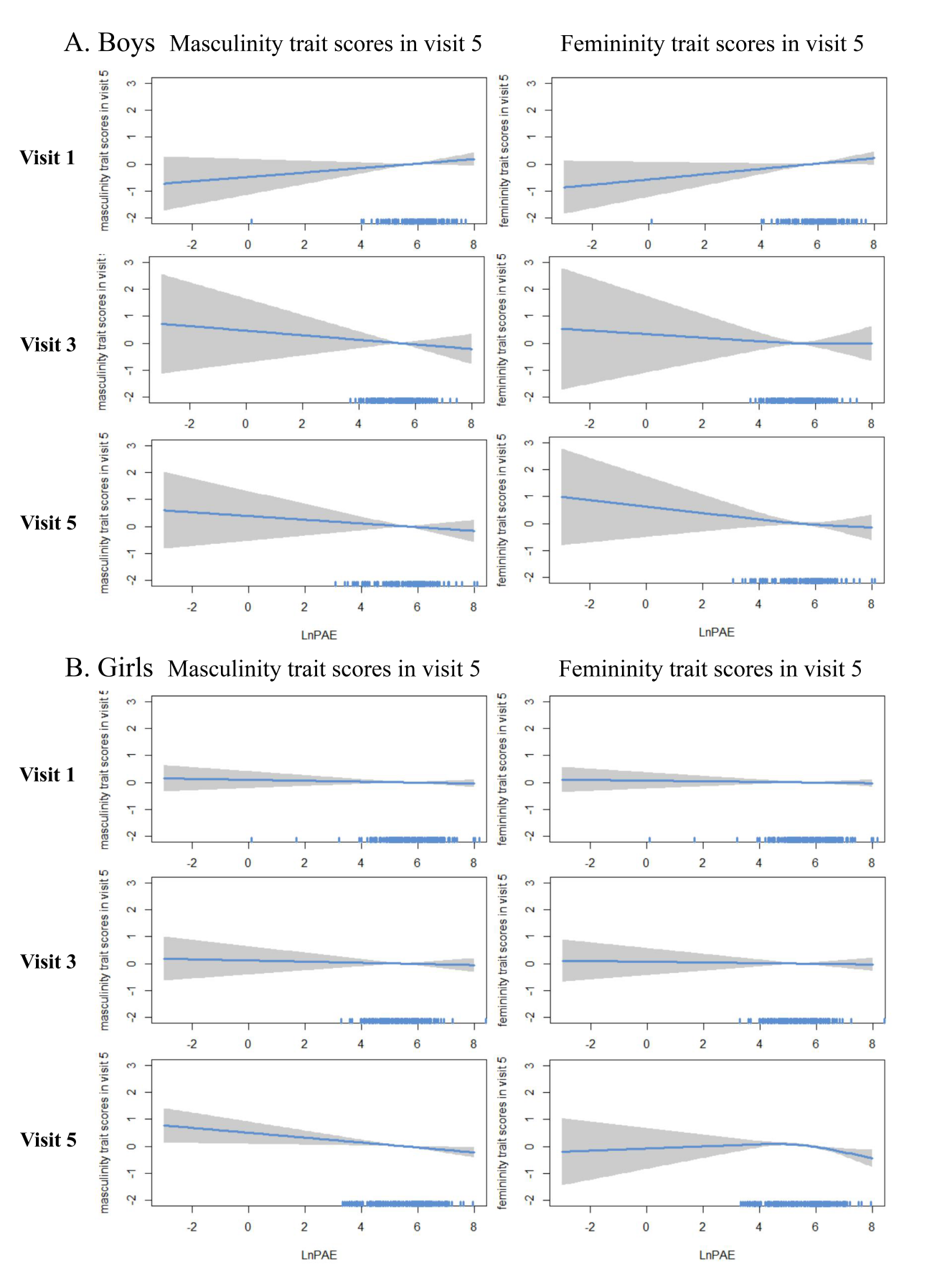


Figure S3. The results of GAM for the total PAEs exposure and the gender indentity scores in visit 5 (A. Boys; B. Girls)


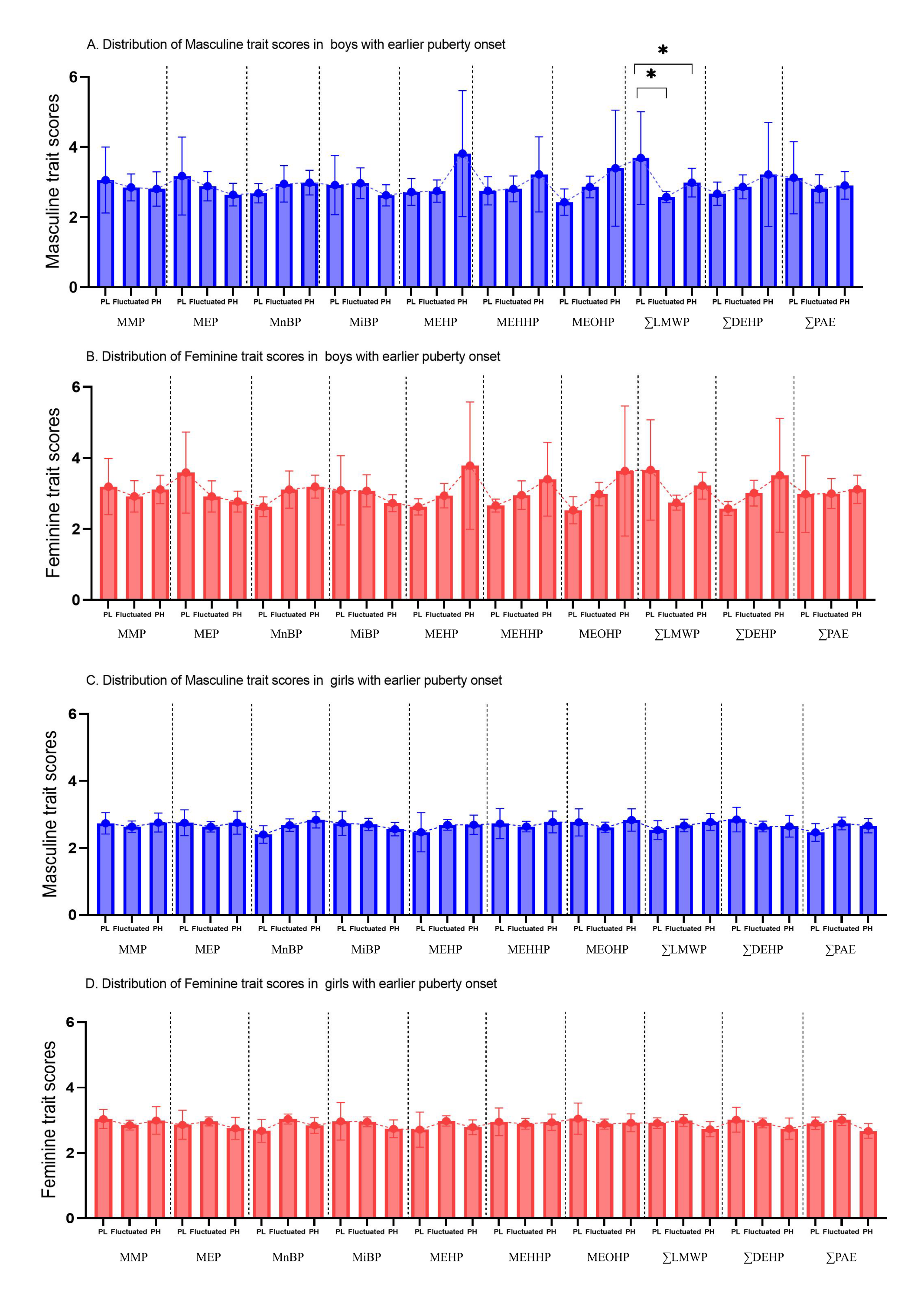


Figure S4. Distribution of gender trait scores in boys and girls with earlier pubertal onset. (*referred to significant differences).


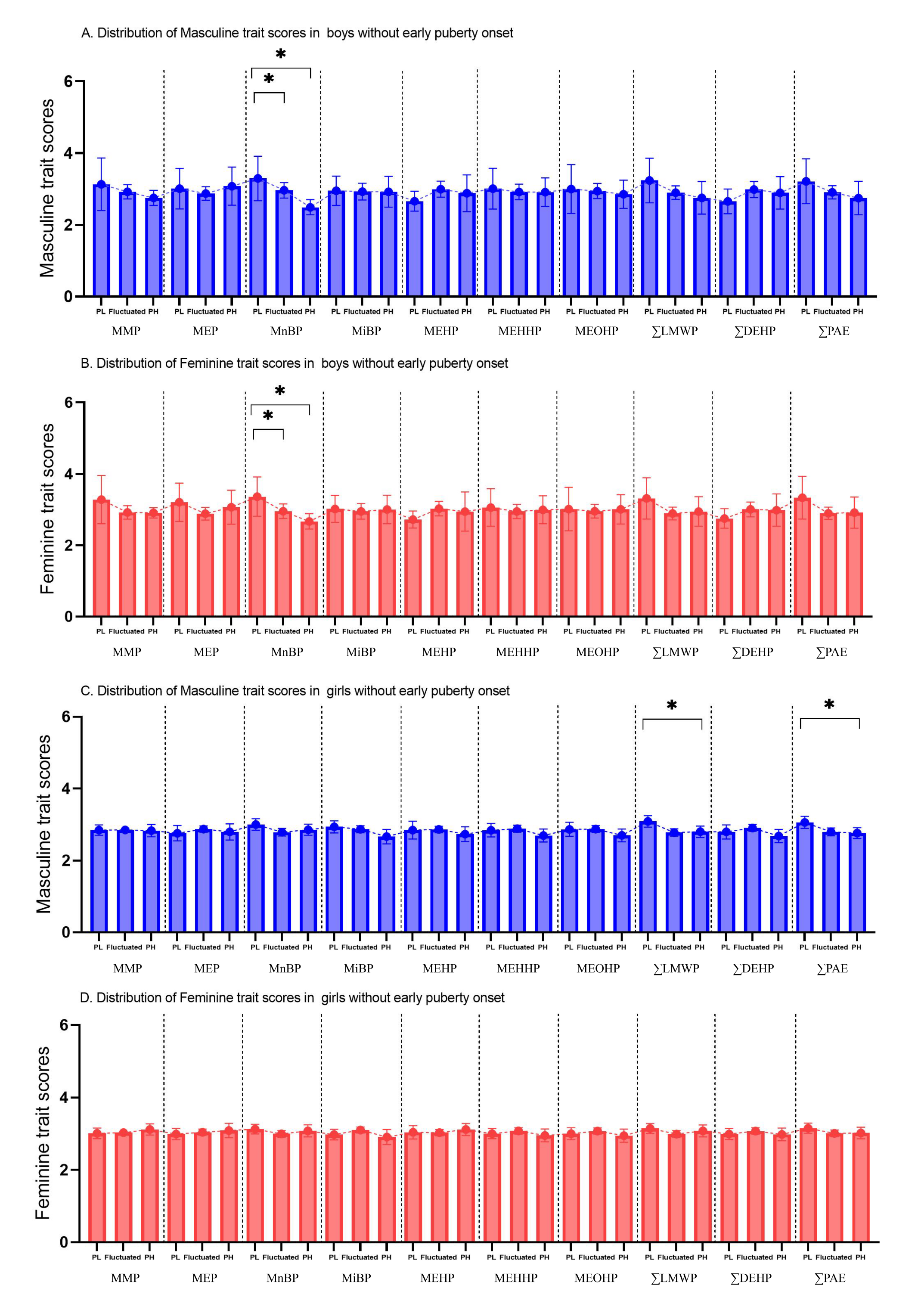


Figure S5. Distribution of gender trait scores in boys and girls without early pubertal onset. (*referred to significant differences).


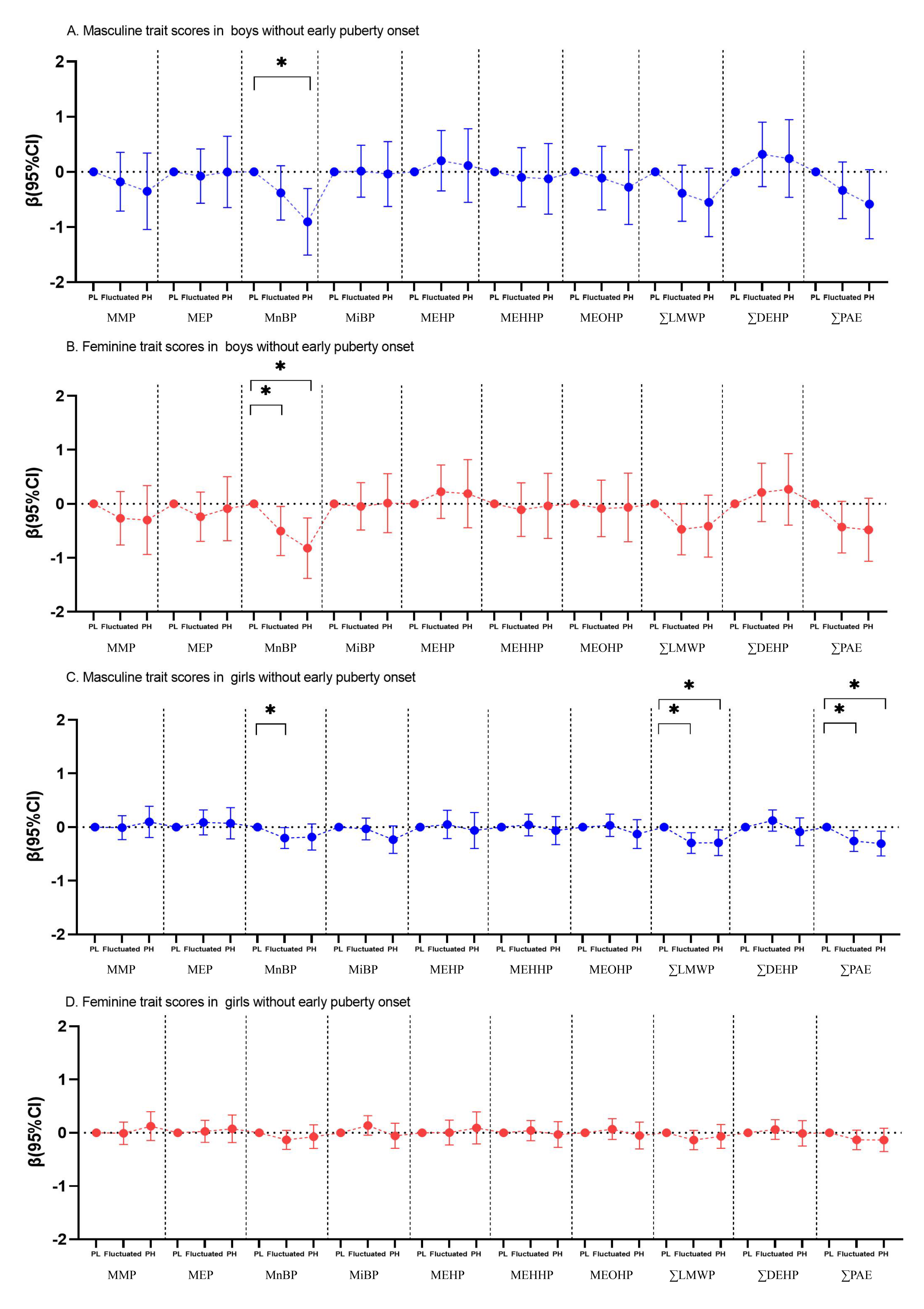


Figure S6. Linear associations between long-term PAEs exposure and the masculinity trait and femininity trait scores in children without early pubertal onset. (*95%CI did not contain 0 referred to *P*<0.05.)

Figure S7. The results of GAM for the total PAEs exposure and the difference of gender indentity scores between visit 1 and visit 5 (A. Boys; B. Girls)


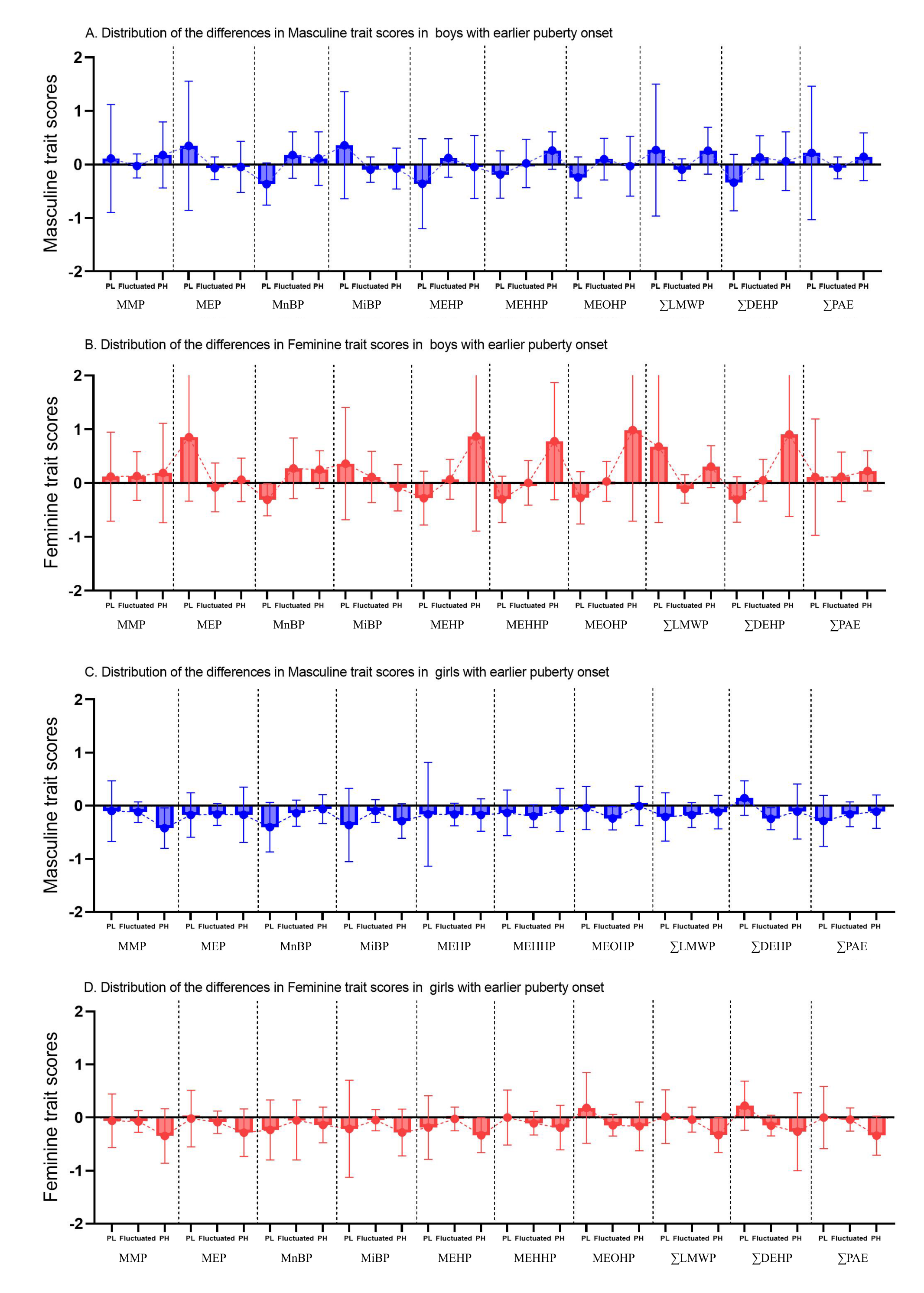


Figure S8. Distribution of the differences in gender trait scores between the visit 1 and the visit 5 in boys and girls with earlier pubertal onset.


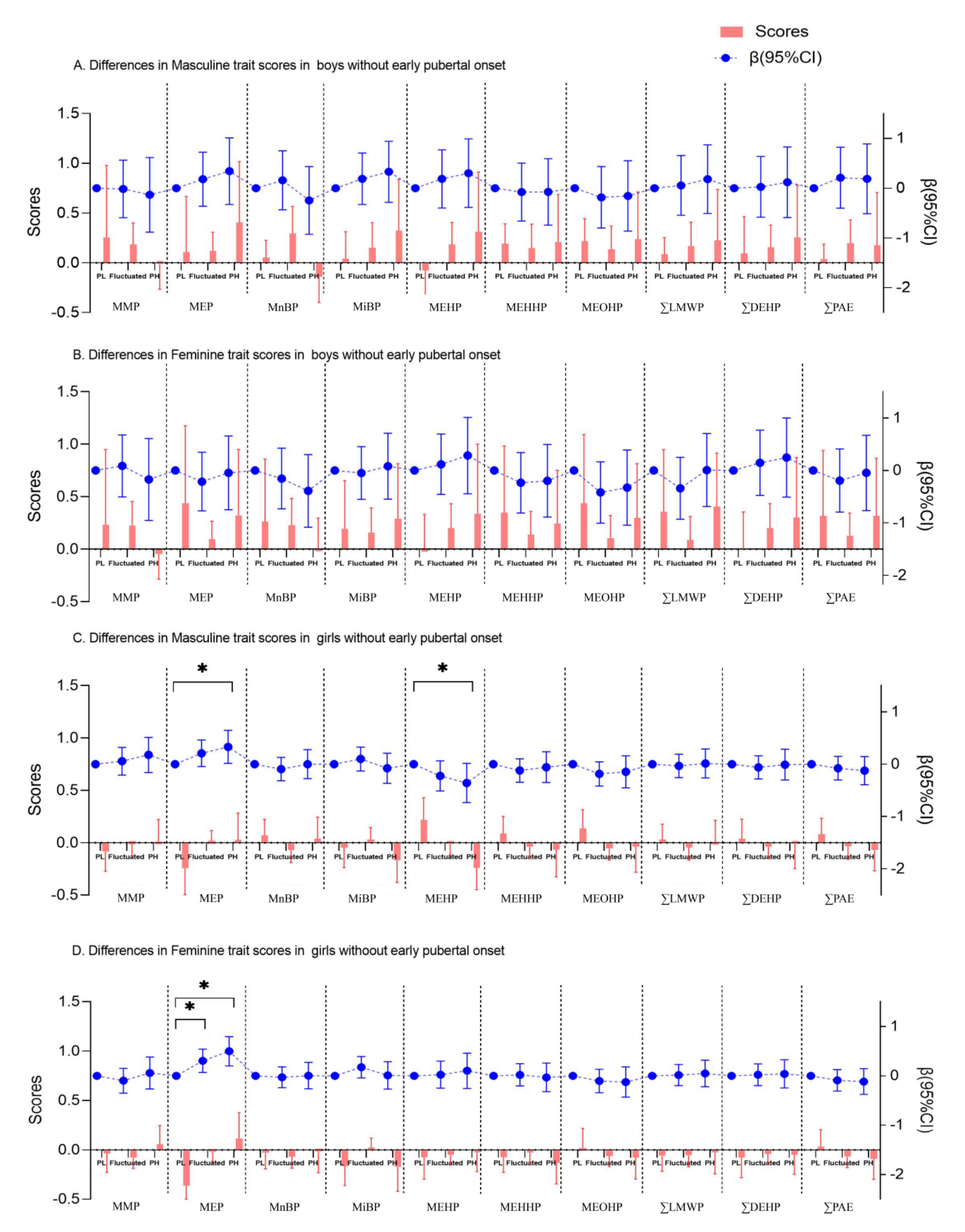


Figure S9. Distribution of the differences in gender trait scores between the visit 1 and the visit 5 and its associations with long-term PAEs exposures in boys and girls without early pubertal onset. (*95%CI did not contain 0 referred to *P*<0.05.)


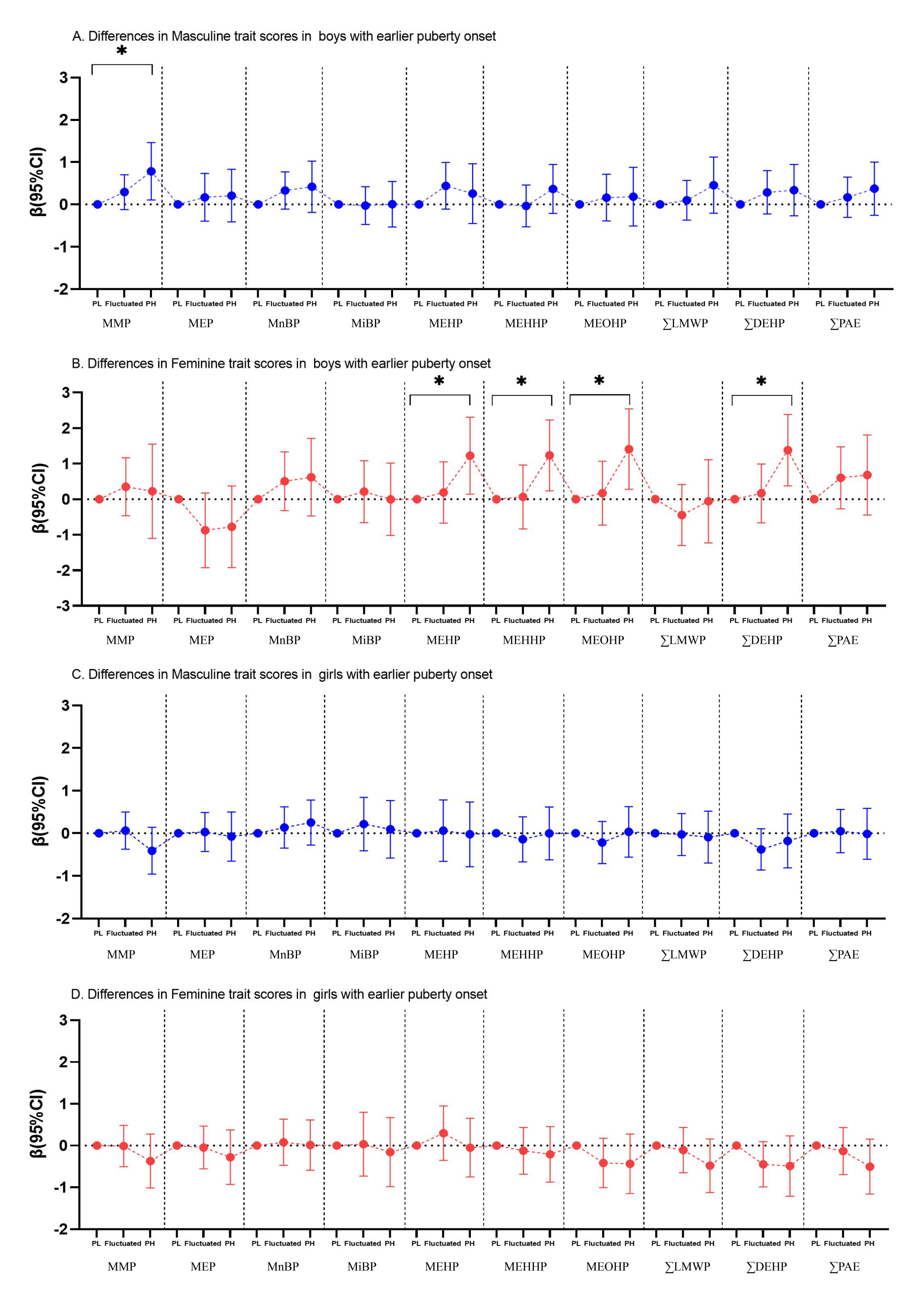


Figure S10. Linear associations between long-term PAEs exposure and the differences in gender trait scores between visit 1 and visit 5 in children with early pubertal onset. (Model was additionally adjusted for BMI. *95%CI did not contain 0 referred to P<0.05.)


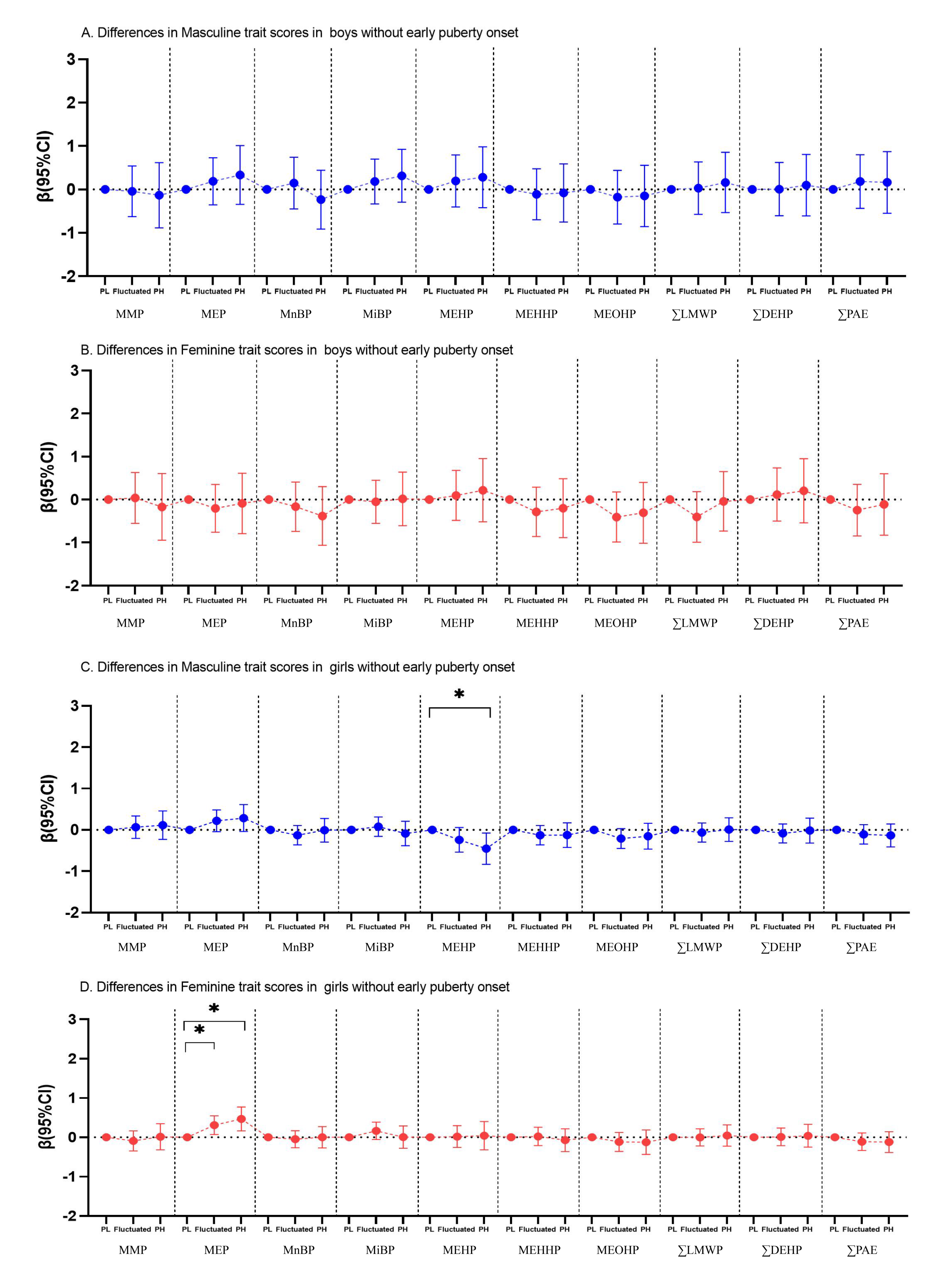


Figure S11. Linear associations between long-term PAEs exposure and the differences in gender trait scores between visit 1 and visit 5 in children without early pubertal onset. (Model was additionally adjusted for BMI. *95%CI did not contain 0 referred to P<0.05.)


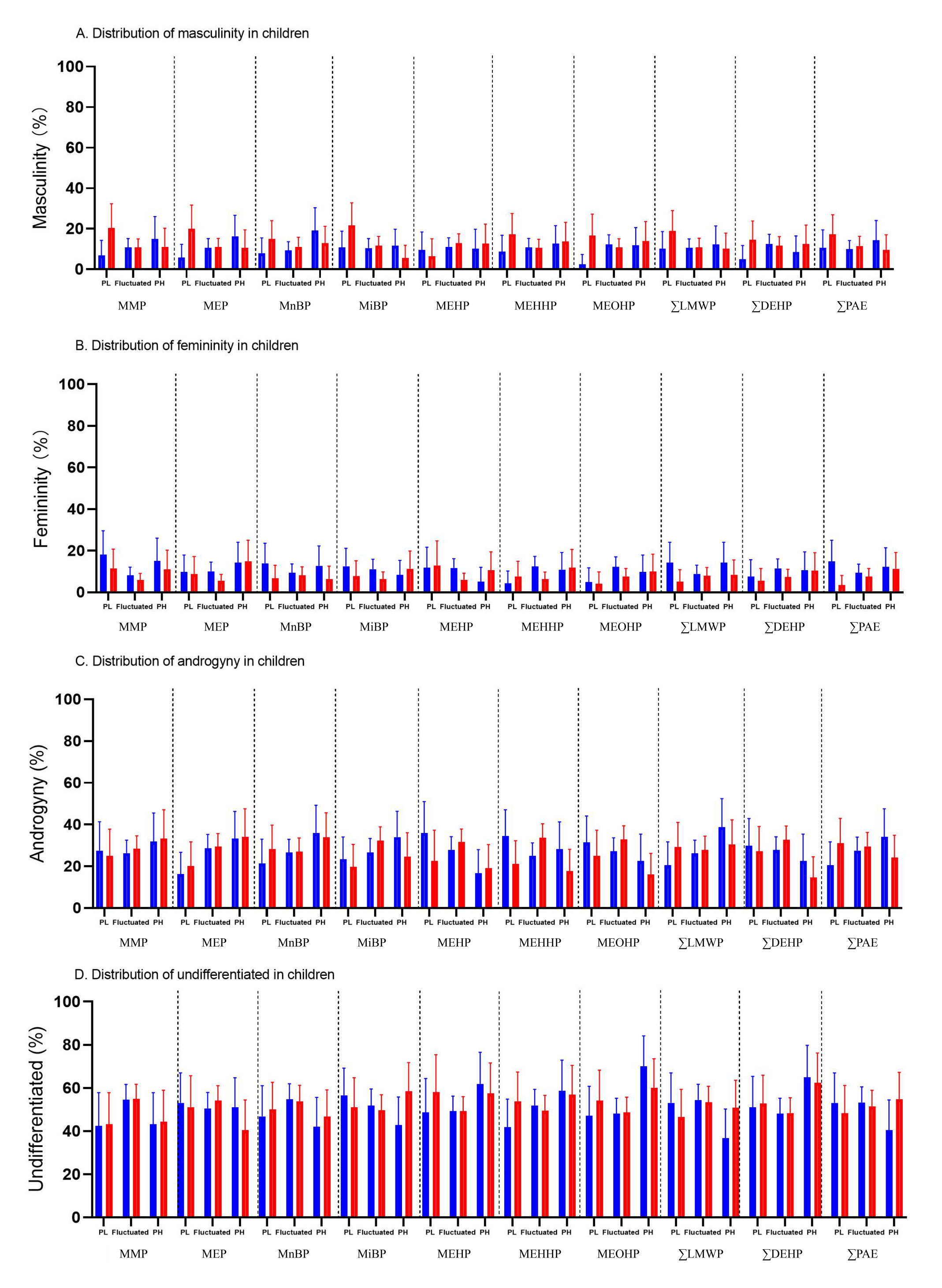


Figure S12. Distribution of gender identity in boys and girls. (Blue referred to boys and red referred to girls).


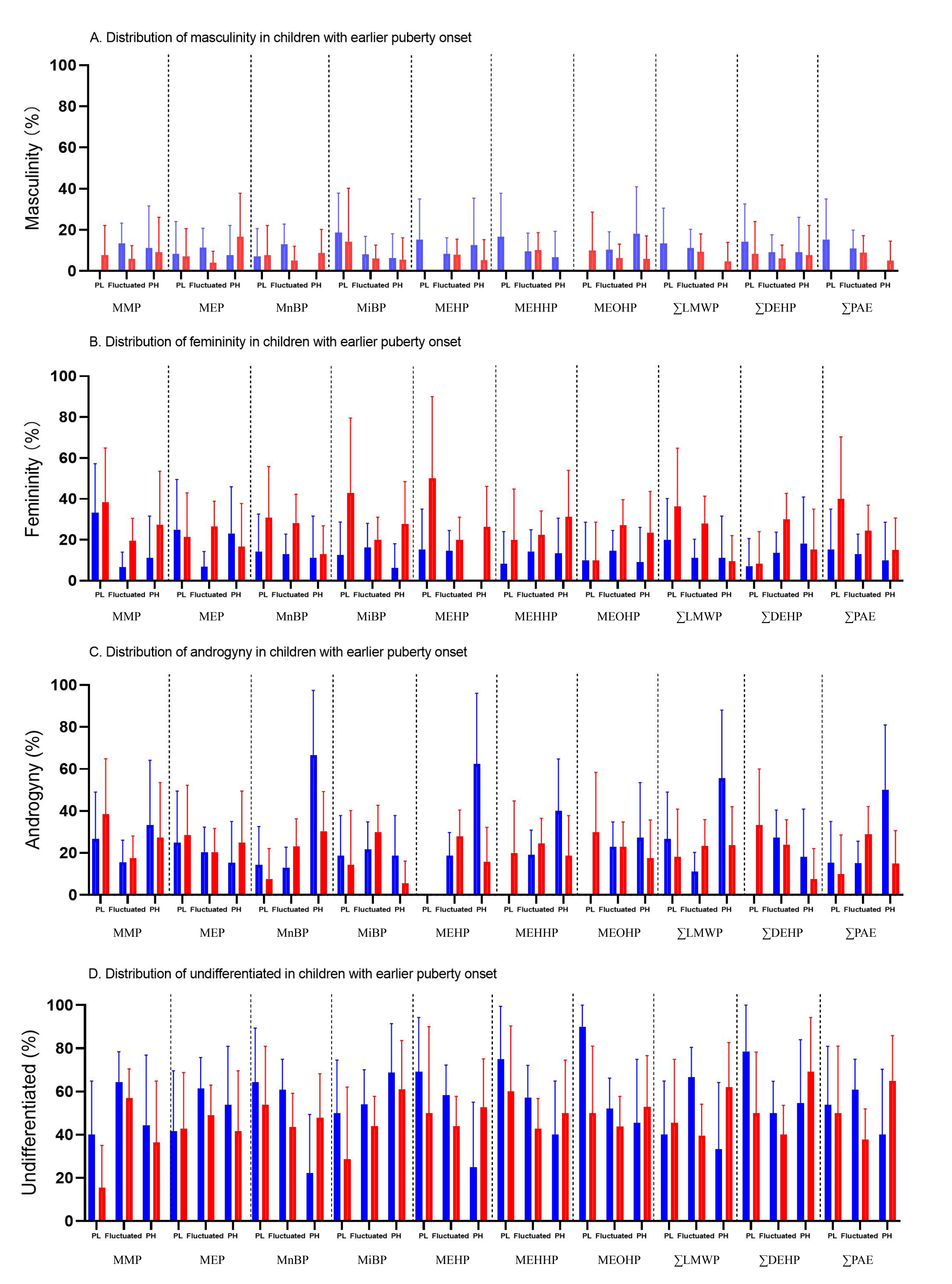


Figure S13. Distribution of four types of gender identity in children who entered puberty earlier. (Blue referred to boys and red referred to girls).
